# ProMol_Func: A Structure-Free Deep Learning Model for Virtual Screening

**DOI:** 10.1021/jacsau.6c00173

**Published:** 2026-02-24

**Authors:** Zixuan Feng, Max Kim, Aweon Richards, Tania J. Lupoli, Yingkai Zhang

**Affiliations:** † Department of Chemistry, 5894 New York University, New York, New York 10003, United States; ‡ Simons Center for Computational Physical Chemistry, 5894 New York University, New York, New York 10003, United States; ¶ NYU−ECNU Center for Computational Chemistry, NYU Shanghai, Shanghai 200062, China

**Keywords:** virtual screening, structure-free, deep learning, protein−ligand binding, zero-shot learning, *E. coli* DnaK

## Abstract

In computational-aided drug discovery, structure-based drug design models are computationally intensive and rely on protein structures, limiting their scalability and generalization. Additionally, many existing models suffer from inflated false-positive rates due to the scarcity of negative binding data for training. To overcome these challenges, we present ProMol_Func, a structure-free deep learning framework that integrates graph-based encodings of small molecules with protein function embeddings derived solely from amino acid sequences. By augmenting the training data set with both experimentally validated inactives and randomly selected decoys, ProMol_Func improves screening power and generalization. The model achieves state-of-the-art performance on the challenging LIT-PCBA (Library of Integrated Targeted-Panel of Cell-Based Assays) benchmark, with an enrichment factor (EF1%) of 10.9, demonstrating robust screening power in realistic assay settings. Furthermore, in a zero-shot prospective application to *E. coli* DnaK, a protein chaperone without actives in the training set, ProMol_Func successfully identified compounds that inhibit its ATPase activity or alter the protein’s thermal stability, validating the potential of ProMol_Func for discovering binders toward novel targets. These results position ProMol_Func as an efficient and scalable alternative to traditional structure-dependent approaches in early stage hit discovery.

## Graphical abstract

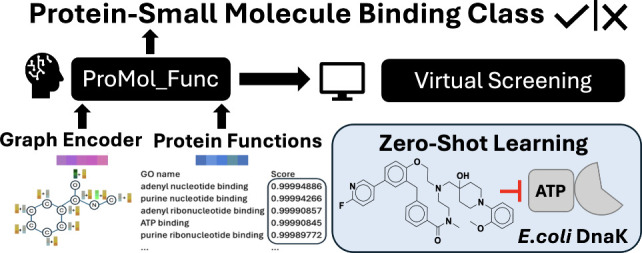

Virtual screening (VS) is a computational technique in drug discovery used to search large libraries of small molecules and identify candidates likely to bind a specific biological target for experimental validation.^1–3^ Docking, a key method in structure-based drug design (SBDD), simulates ligand–receptor binding by evaluating geometric fit and binding energy, often achieving higher hit rates than high-throughput screening (HTS).^1,4–7^ Its success relies on sampling diverse ligand conformers to find optimal binding orientations and accurately estimating binding affinity using scoring functions.^5,8–17^ The extensive conformational sampling leads to significant computational demands, making docking tools less practical for large-scale VS,^1^ particularly in the early phases of drug discovery, where speed and efficiency is crucial. Meanwhile, SBDD models face more challenges when applied to novel targets lacking high-quality crystal structures or targets without known or well-resolved binding sites.^1,18,19^

In recent decades, machine learning (ML) has significantly advanced the field of drug discovery.^2,24–30^ However, data-driven deep learning models trained on PDBbind structures for regression tasks such as binding affinity prediction, lack negative data to enforce the training, thus their enrichments of true hits are limited or biased.^2,31,32^ Many ML-driven hit discovery methods with experimental validation^33–35^ remain target-specific and struggle to generalize beyond known binders. Recently, BIND^36^ introduced a structure-free deep learning framework that represents compounds as molecular graphs derived from SMILES and encodes proteins using ESM-2,^37^ a state-of-the-art protein language model. Although BIND achieved superior performance on well-established virtual screening benchmark test sets, it has not been prospectively validated experimentally, and thus the real-word applicability of structure-free deep learning approaches has not been demonstrated.

In realistic early stage hit discovery scenarios, researchers often begin without experimentally confirmed binders for a novel protein target while several binders may have already been found for homologues, proteins with similar molecular functions (MF).^38,39^ Considering that small molecules often exert their effects by modulating MF, either directly or allosterically,^40–42^ here we were motivated to explore a function-level modeling strategy in which protein molecular function embeddings serve as input features in binder classification. This enabled structure-free virtual screening by bypassing the need for 3D structural information altogether. To represent proteins, we used DeepFRI^20^ to predict MF from sequence alone, enabling applicability to proteins without structural data. These function scores were linearly transformed and added to trainable small molecule graph embeddings generated by KANO,^22^ as outlined in the ProMol_Func framework shown in Figure 1. Later we evaluated ProMol_Func’s zero-shot learning ability on a target chaperone protein, *E. coli* DnaK, and experimentally validated two ligands and one destabilizer.

**Figure 1:**
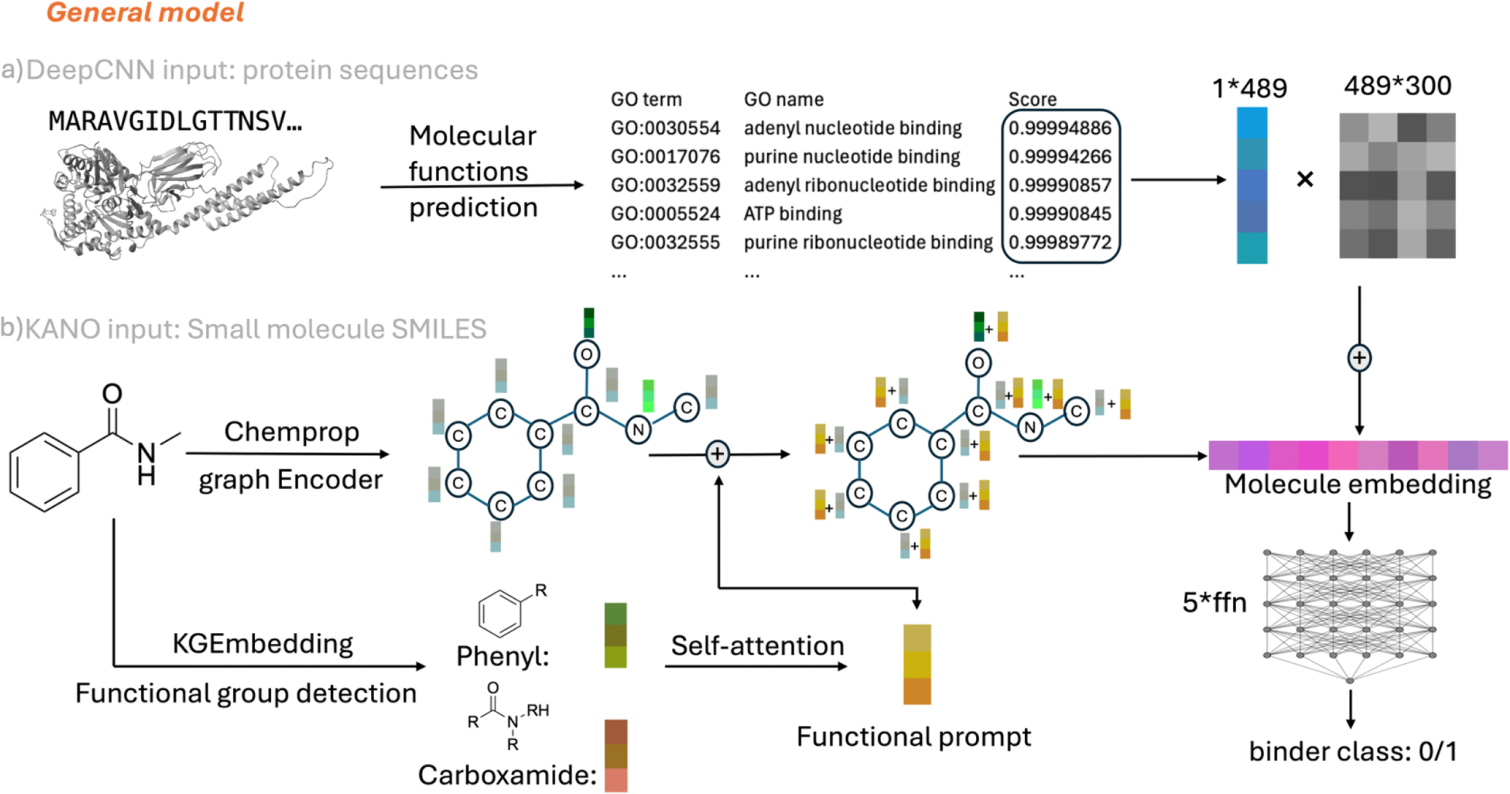
ProMol_Func incorporates a pretrained sequence-based protein function prediction model and a 2D graph encoder for small molecules to train a general classification model to prioritize binders over nonbinders. a) DeepCNN^20^ takes protein sequences as input (the structure shown is an AlphaFold2 ^21^ prediction of DnaK based on the GenBank CAA41306 sequence), and 489 classes of protein molecular function prediction scores are transferred as protein embeddings and combined with b) KANO^22^ small-molecule representations, followed by feed-forward layers for binary classification. In KANO^22^ architecture, functional prompts were added to the Chemprop^23^ graph encoder. The KANO model schematic is adapted from ref 22, licensed under CC BY 4.0. All other aspects of the figure are original.

## Model Development and Evaluation

Results on a pilot data set showed that adding protein function embeddings during training slightly outperforms concatenation-merging two vectors (Table S1). To expand the training data, we extracted 2.55 M active protein–ligand pairs covering 7445 proteins from BindingDB.^43^ Each pair was annotated with at least one of the following binding affinity metrics, K_*I*_, IC_50_, EC_50_ or K_*D*_, with values below 50 μM (Scheme S1). Guided by large-scale analyses of virtual screening campaigns^44^ and the potency distribution of LIT-PCBA actives,^45^ which show that most hits are reported in the low- to midmicromolar range, we adopted 50 μM as a practical and reproducible cutoff to define actives and inactives (see Supporting Information). Accordingly, 0.19 M compounds with any of the metrics higher than 50 μM were labeled as inactives. In addition, 1.30 M inactives (2000 inactive compounds per protein for around 650 proteins) from Pubchem^46^ bioassays were retrieved. To address data imbalance, we additionally sampled 2.55 M decoys, referring to assumed negatives, from BindingDB (details in SI). In total, the curated ProMol_Func data set includes 7817 proteins and 6.60 M compounds. To enhance robustness and generalization, we trained an ensemble of three models with different scaffold-balanced splitting seeds. This ensemble, referred to as ProMol_Func, with predictions averaged, was used for downstream benchmarking evaluations.

LIT-PCBA (Library of Integrated Targeted-Panel of Cell-Based Assays)^45^ is an unbiased subset of the broader PubChem BioAssay data consisting of 15 protein targets and is widely used for virtual screening and drug discovery. ProMol_Func achieved a top average 1% enrichment factor (EF1%) of 10.90 across all LIT-PCBA targets (Figure 2), closely matching the best-performing model, BIND (EF1% = 10.93). This demonstrates ProMol_Func’s superior screening power in real-world scenarios. Per-target EF values are reported in Table S2, which also illustrates that certain targets (e.g., ESR1_ago and MTORC1) remain more challenging and provides a detailed view beyond the averaged results in Figure 2. For overlap analysis, we found highly similar protein–compound pairs between the training data and LIT-PCBA (Table 1), with per-target counts summarized in Table S3. However, after removing the 209 identical compounds from LIT-PCBA, the ProMol_Func EF1% remained 10.90. Furthermore, after excluding samples whose protein-small-molecule similarity scoring (methods in SI) exceeded thresholds of 0.9, 0.7, or 0.5 from LIT-PCBA, ProMol_Func EF1% remain consistently strong for “dissimilar” samples as shown in Table 1, with per-target EF1% results provided in Tables S4–S6.

**Figure 2:**
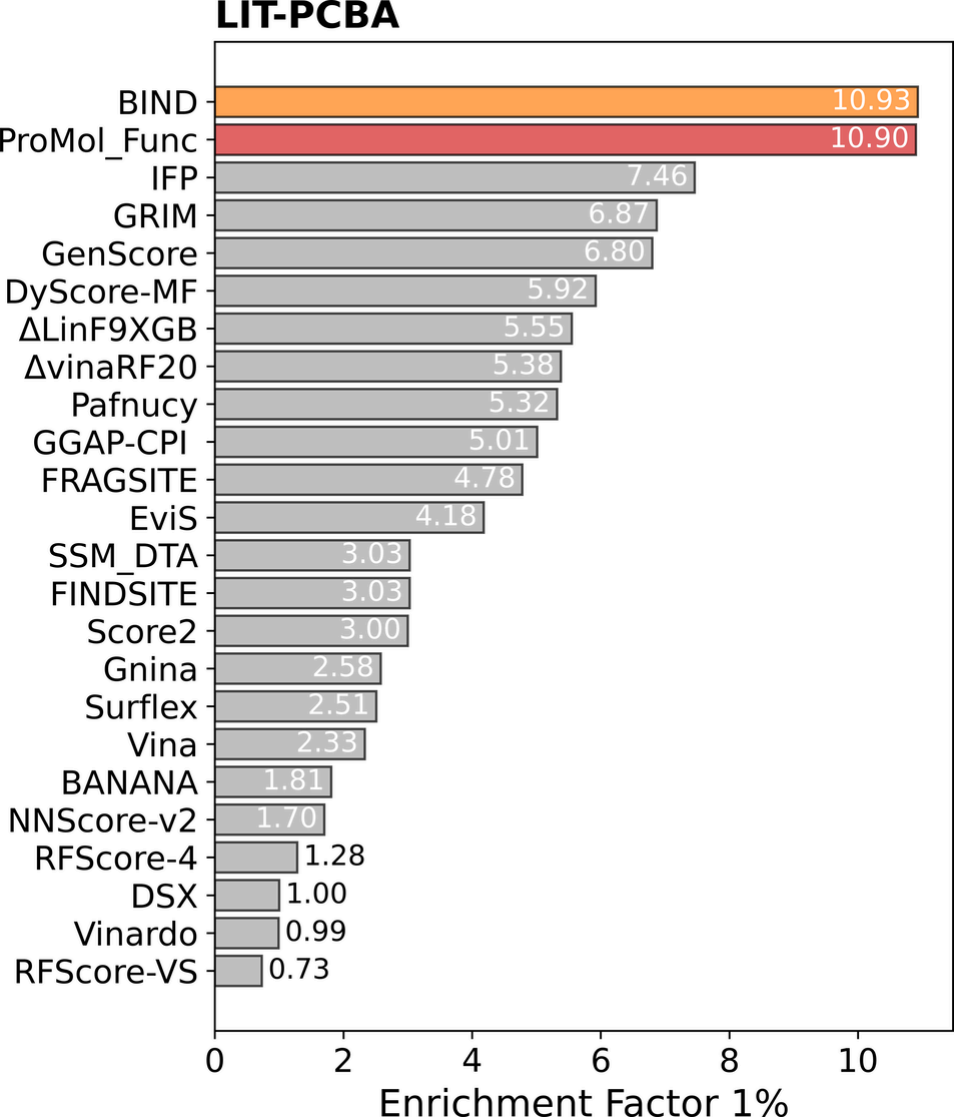
Top 1% enrichment factor (EF1%) for the ProMol_Func general model (ensemble predictions of 3 models) on LIT-PCBA targets. Performance of other models is reported based on data from BIND^36^ and previous work.^47,48^ Figure adapted from ref 36 available under a Creative Commons CC BY license.

**Table 1:** ProMol_Func EF1% on the LIT-PCBA Test Library after Removing Identical Compounds and Highly Similar Protein–Compound Pairs (Similarity Thresholds of 0.9, 0.7, and 0.5) from LIT-PCBA^a^

Sim_Threshold	Samples Removed	EF1%
Identical	209	10.90
0.9	15,036	10.89
0.7	31,580	10.76
0.5	135,369	8.91

a “Sim_Threshold” refers to protein sequence identities × small molecule Morgan-fingerprints Tanimoto similarity. “Samples Removed” indicates the number of protein–compound pairs excluded for exceeding the corresponding similarity threshold.

Unlike LIT-PCBA, the DUD-E^49^ and DEKOIS2.0 ^50^ data sets incorporate decoys. On DUD-E, ProMol_Func achieved the highest EF1% (48.67), surpassing BIND (46.35),^16^ while ranking second on DEKOIS2.0 with an EF1% of 22.94 (Figure S2). ProMol_Func also demonstrated consistently strong EF1% on the “dissimilar” subsets of both DUD-E and DEKOIS2.0 (Tables S7 and S8). These results confirm the robustness and generalizability of ProMol_Func in virtual screening tasks for various proteins.

Negatives data are essential for building robust classification models.^36,51^ As shown in Table 2, the inclusion of decoys significantly enhances our model’s predictive performance. Model 1, trained on 1.02 M actives and 1.20 M inactives, achieved an EF1% of only 3.24 on the LIT-PCBA benchmark. Introducing 1.02 M decoys significantly improved the EF1% to 6.83. This improvement likely stems from the imbalance between inactives and actives for individual proteins (data distribution plotted in Figure S1), which decoys help to balance

**Table 2:** Training Data Size and Top 1% Enrichment Factor (EF1%) on the LIT-PCBA Benchmark for Different Promol_Func Models

Model	Actives	Inactives	Decoys	EF1%
Model 1	1.02 M	1.20 M	0	3.24
Model 2	1.02 M	1.20 M	1.02 M	6.83
Model 3	2.04 M	1.20 M	2.04 M	8.22
3*Model 3^a^	–	–	–	10.90

a 3*Model 3 means an ensemble of three Model 3 with averaged predictions and is referred to as *ProMol_Func*.

## Zero Shot Learning Application

Heat shock protein families, namely HSP90 and HSP70, are ATP-dependent chaperones that maintain proteostasis in cells by folding and stabilizing client proteins or assisting in targeting proteins for degradation. Eukaryotic HSP70s and HSP90s have emerged as synergistic targets in anticancer therapy.^52–55^ The training data included many actives and inactives for human HSP90 (Uniprot ID: P07900). Despite a highly imbalanced human HSP90 external test set (∼30 times more inactives; Table 3), ProMol_Func achieved strong accuracy with an F1 score of 0.858 and precision of 0.941 at a 0.5 threshold (Table S9).

**Table 3:** Overview of Active and Inactive Data for human HSP90 and *E. coli* DnaK^a^

	Training Data set		Test Data set	
Target	Actives	Inactives	Actives	Inactives
HSP90	2658	9411	1579	284049
ecDnaK	0	24	24	3634

a External data for human HSP90 was collected from Pubchem AID 1789, 1946, 1947, 1912, 1913 and chemBL, D3R AbbVie-CSAR. External *E. coli* DnaK (ecDnaK) data was collected from Pubchem AID 1033 and previous works.^56–58^ Overlapping data were removed for a model test.

*E. coli* DnaK, a member of the HSP70 family^59–61^ that shares 49.60% sequence identity (global alignment with Clustal Omega via UniProt Align) with a major human HSP70, HSPA1A, has been used as a model for human HSP70 inhibitor identification.^57^ Further, bacterial DnaKs are putative infectious disease targets.^38,62^ However, *E. coli* DnaK has few known binders, limiting target-specific model training. Additionally, its available crystal structures are also scarce and often incomplete, typically representing only individual domains, the nucleotide-binding domain (NBD) or the substrate-binding domain (SBD).^63–65^ *E. coli* DnaK contains multiple allosteric sites and exhibits distinct conformations in ATP- and ADP-bound states,^64,66^ complicating structure-based drug design (SBDD) efforts. The hit rate for HSP70 is known to be lower than for HSP90 in HTS, likely due to HSP70s' tighter nucleotide binding, which makes competitive inhibition more difficult.^67,68^ Additionally, chaperone inhibitors often show only micromolar potency and incomplete inhibition.^38,69^

The training data of ProMol_Func did not include any active compounds for *E. coli* DnaK (Table 3), making it an ideal target of zero-shot learning. Twenty-four active ligands and 3634 inactives from Pubchem and previous works^56–58^ were used as the test set for evaluation (Table 3). ProMol_Func achieved a precision of 0.381 at a 0.45 threshold and 0.143 at 0.5 on the *E. coli* DnaK test set (Table S9), highlighting the model’s ability to transfer across functionally similar proteins while not relying on high sequence identity or structural homology. Despite the low sequence identity between Human HSP90 and *E. coli* DnaK (20.86%, global alignment with Clustal Omega via UniProt Align), their functional similarity may still offer transferable signals. As shown in Figure 3, increasing the threshold reduces false positives in DnaK inhibitor selection. We applied ProMol_Func to screen the commercially available 575302 compounds from the Asinex library for potential inhibitors of *E. coli* DnaK (Uniprot ID: C3TRK2) and identified 185 compounds with predicted binding probabilities above 0.5. From these, 43 compounds were selected and purchased based on their predicted binding probability as well as favorable solubility profiles (Scheme S2).^70^

**Figure 3:**
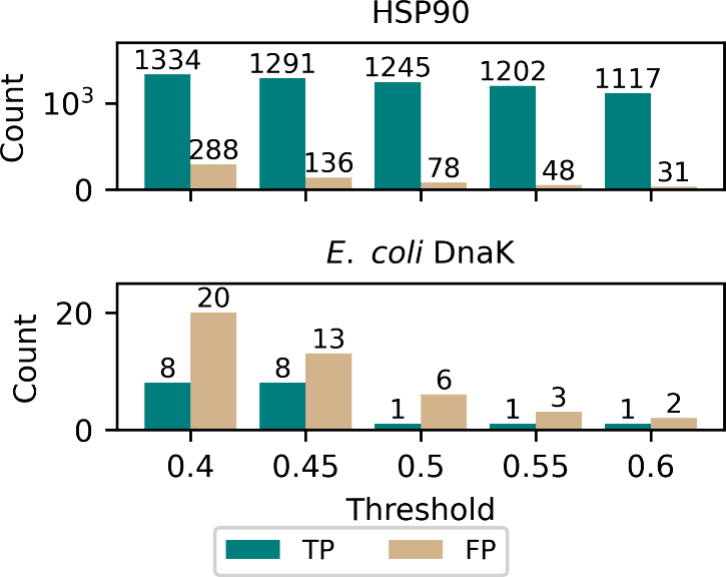
Count of true positives (TP) and false positives (FP) predicted by ProMol_Func on human HSP90 and *E. coli* DnaK (ecDnaK) test data sets across different thresholds.

DnaJ and GrpE are essential cofactors that modulate the ATPase and folding activities of DnaK, as illustrated in Figure 4a. DnaJ recognizes and binds unfolded proteins, subsequently delivering them to DnaK while stimulating its ATPase function.^73^ GrpE acts as a nucleotide exchange factor, promoting the exchange of ADP for ATP and facilitating substrate release.^74^ Together, DnaJ and GrpE tightly regulate DnaK’s ATP hydrolysis, which is critical for DnaK’s chaperone functions.^75,76^ Among the 43 ProMol_Func-predicted binders (EG) of DnaK, EG36 emerged as a promising hit, showing 20–60% inhibition of DnaK ATPase activity stimulated by DnaJ-GrpE. A structural analog, EG35, also showed inhibition, but with weaker a potency (Figures 4b and S3). Co-sedimentation experiments^77^ confirmed that the compounds do not aggregate under ATPase assay conditions (Figure S4). Both exhibited modest potency relative to Telaprevir (TP),^38^ an HSP70 SBD inhibitor that allosterically reduces ATP turnover (Figure 4c). Notably, EG36 showed stronger inhibition of ADP production with increasing [DnaJ], suggesting it may disrupt functional DnaK–DnaJ interactions (Figure 4d).

**Figure 4:**
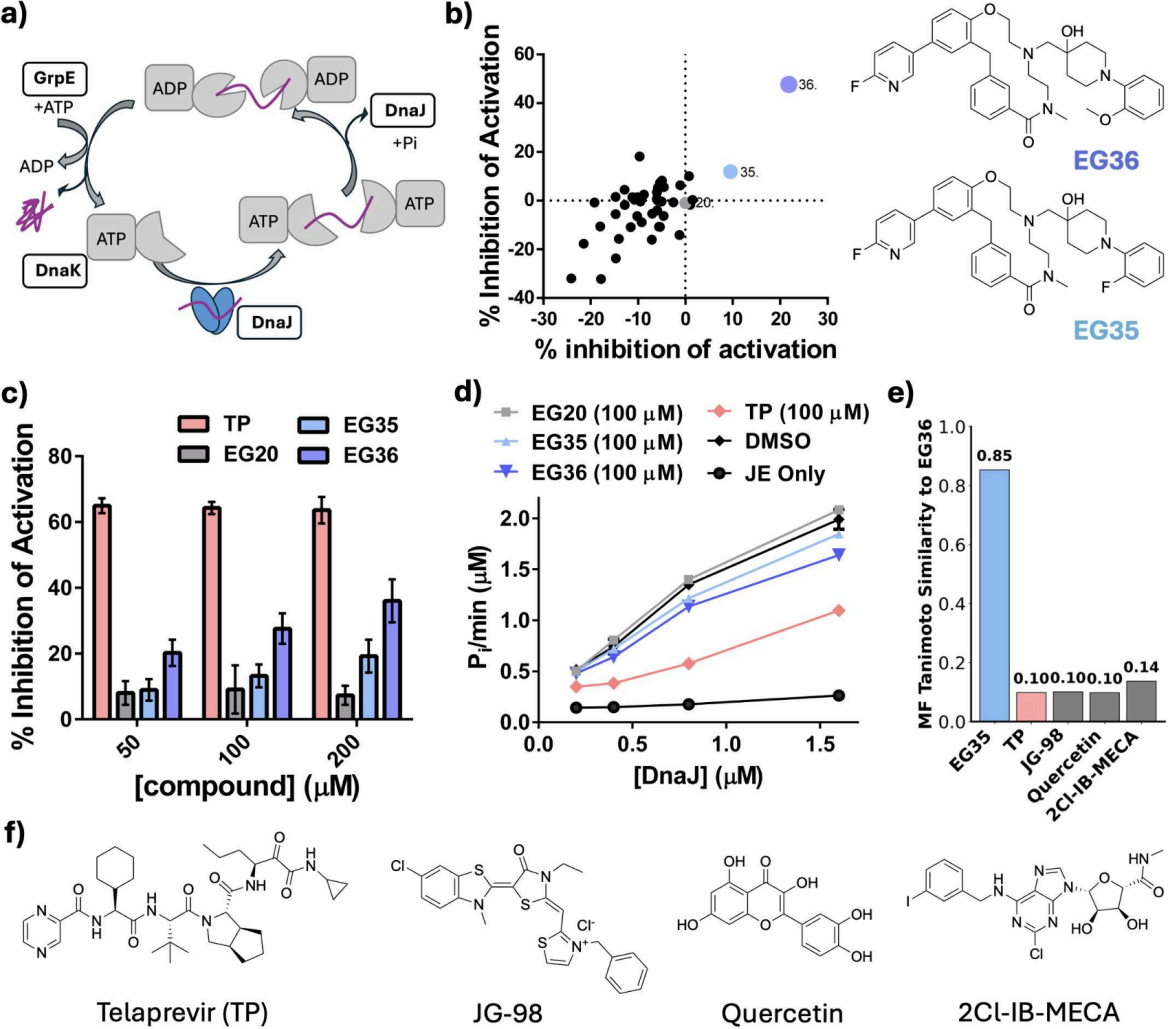
a) Scheme of cofactor-mediated (DnaJ and GrpE) activation of *E. coli* DnaK’s ATPase activity.^71^ b) Of the 43 compounds (100 μM) predicted by ProMol_Func to bind *E. coli* DnaK, two (EG35 and EG36) showed consistent inhibition of ATPase activity over two trials; chemical structures of hit compounds on the right (100 μM compound, 4 μM *E. coli* DnaK + 0.4 μM each of DnaJ2 and GrpE). c) Titration of EG35 and EG36 to DnaK-cofactor ATPase reactions (4 μM *E. coli* DnaK and 0.4 μM DnaJ2 and GrpE) indicates inhibition. Known inhibitor telaprevir (TP)^38^ serves as a positive control. EG20 did not show inhibition, thus acting as a negative control. d) Compounds’ inhibition of DnaK (4 μM) and GrpE (0.4 μM) reactions with increasing DnaJ. e) Morgan fingerprints Tanimoto similarity of EG36 to EG35 and other known *E. coli* DnaK inhibitors. f) Chemical structure of known DnaK inhibitors: Telaprevir (TP),^38^ JG-98,^72^ Quercetin^56^ and 2Cl-IB-MECA.^38^

Notably, EG36 exhibits high structural similarity (0.85) to EG35 yet displays low similarity to known DnaK inhibitors (lower than 0.2) such as TP,^38^ JG-98,^72^ Quercetin^56^ and 2Cl-IB-MECA^38^ (Figures 4e,f and S5), underscoring their structural novelty. Fluorescence polarization (FP) assays showed that neither EG36 nor EG35 displaced the fluorescent peptide bound to the SBD, unlike TP, indicating they do not target the peptide-binding cleft (Figure S7). AlphaFold3 ^78^ predicted EG36 binding at an allosteric site on the NBD, while Boltz-2 ^79^ suggested interaction at the NBD-SBD linker domain (Figure S9). Both compounds inhibited the ATPase activity of the isolated NBD, supporting direct binding to this domain (Figure S10). Collectively, these results suggest that EG36 and EG35 represent a novel class of NBD-targeting inhibitors. Furthermore, they do not appear to be general Hsp inhibitors, as neither affected the thermal stability nor ATPase activity of a bacterial HSP90 called HtpG^80^ (Table S12, Figure S11). Nonetheless, their moderate inhibition of DnaK–cofactor ATPase activity indicates room for further optimization.

We next assessed the impact of ProMol_Func-predicted candidates on *E. coli* DnaK thermal stability using nanoDSF, a tagless thermal denaturation method (Figure S6).^81,82^ While ligands like TP stabilized DnaK and increased its melting temperature (*T*_m_) by ∼1.2 °C, known inhibitors quercetin and 2Cl-IB-MECA reduced the *T*_m_ by <1.0 °C, suggesting minimal destabilization (Table S10). Similarly, EG35 and EG36 did not cause a noticeable change in *T*_m_ (Table S11, Figure S6), likely because they interact with an allosteric and shallow cleft and do not substantially perturb the thermal stability of the protein.^83–85^ In contrast, EG31 (500 μM) reduced the *T*_m_ by ∼1.5 °C (Figures S6 and S8), possibly by disrupting DnaK oligomerization.^86^ However, EG31 did not affect cofactor-stimulated ATPase activity, suggesting its binding is either disrupted by cofactors or occurs outside the ATPase-regulating site.

In summary, we present ProMol_Func, a generalizable framework by integrating sequence-derived protein function embeddings as global conditioning into trainable small-molecule graph encoders to predict protein–compound binding probabilities. ProMol_Func demonstrates strong screening power across multiple benchmarks, outperforming advanced SBDD models and rivaling the state-of-the-art structure-free model BIND. While we utilized 2.60 M trainable parameters, BIND operated with 9.26 M, so ProMol_Func has lower model complexity. Incredibly, ProMol_Func finished training on 5.3 M protein–ligand pairs for 9–10 epochs during 3–4 days on a single Nvidia A100 GPU. Importantly, ProMol_Func demonstrates zero-shot learning capability by successfully identifying novel binders for *E. coli* DnaK. Experimental validation confirmed this performance: two predicted compounds inhibited bacterial DnaK ATPase activity, and one compound destabilized DnaK, supporting the model’s reliability in prospective applications. We predict that the two inhibitors bind an allosteric NBD site, showing micromolar affinity and partial inhibition, consistent with typical chaperone inhibitor potencies.^38,69^ In the future, structural characterization will be pursued following hit-to-lead optimization. These findings highlight ProMol_Func as a scalable, structure-free tool for early stage hit discovery in virtual screening, especially for targets lacking structural or biochemical data. However, since zero-shot learning is not universally guaranteed to perform well across all targets, users should carefully assess ProMol_Func’s performance on specific systems before relying on its zero-shot predictions.

## Supplementary Material



## Data Availability

Corresponding source code is available at https://github.com/ZixuanFeng-NYU/ProMol_Func. Data and saved model checkpoints are available at 10.5281/zenodo.16825387.

## Notes

Additional figures and tables for extended benchmark results and experimental supporting assays (PDF)
