# Supplementary material for: ProMol_Func: A Structure-Free Deep Learning Model for Virtual Screening
Source: JACS Au. 2026 Feb 24;6(3):1495–502. doi: 10.1021/jacsau.6c00173 (PMC13014237; doi:10.1021/jacsau.6c00173)
Supplement: Supplementary file 1 [file au6c00173_si_001.pdf]

Supporting Information: ProMol_Func: A Structure-Free Deep Learning Model for Virtual Screening

Zixuan Feng,[†,‡] Max Kim,[†] Aweon Richards,[†] Tania J. Lupoli,[*,†] and Yingkai Zhang[*,†,‡,¶]

†*Department of Chemistry, New York University, New York, NY 10003, USA*

‡*Simons Center for Computational Physical Chemistry, New York University, New York, NY 10003, USA*

¶*NYU–ECNU Center for Computational Chemistry, NYU Shanghai, Shanghai 200062, China*

*E-mail: tjl229@nyu.edu; yingkai.zhang@nyu.edu

Table S1: To evaluate addition versus concatenation of DeepFRI protein function embeddings with KANO embeddings, we first used a small-size pilot dataset from BindingDB (<10 nM for actives, >25 µM for inactives). 3 layers of feed forward network were used in this part. a) data composition (sampled from BindingDB); b) data scaffold-balanced splitting for training : validation : test; c) model parameters and performance on validation and test sets. In contrast, the subsequent training of ProMol_Func employed a broader dataset with a universal 50 µM threshold.

(a) Data composition

**Class**	**IC50**	**#pairs**	**Percentage**
1	<10nM	247174	56.85%
0	>25µM	187480	43.15%

(b) Data splitting

**Total**	**Train size**	**Val size**	**Test size**
434147	347317	43414	43416

(c) Model parameters and performance

**Methods**	**Model parameters**	**Validation accuracy**	**Test accuracy**
add	2,325,806	0.939006	0.939400
concat	2,415,806	0.929147	0.934149

Table S2: Target name, corresponding PDB code, and enrichment factors (EF0.5%, EF1.0%, EF5.0%, EF10.0%) based on ProMol_Func predictions for each target in the LIT-PCBA library.

**Target Name**	**PDB Code**	**EF_0.5%**	**EF_1.0%**	**EF_5.0%**	**EF_10.0%**
ADRB2	3p0g	11.76	11.76	3.53	1.76
ALDH1	4wp7	2.09	1.99	1.73	1.68
ESR1ago	1l2i	0.00	0.00	4.62	4.62
ESR1ant	1xp1	3.92	2.94	1.57	1.18
FEN1	5fv7	24.93	17.34	5.47	3.39
GBA	2v3d	4.82	2.41	2.05	1.69
IDH1	4i3k	25.64	12.82	4.62	2.82
KAT2A	5h84	12.37	10.31	4.64	3.14
MAPK1	1pme	5.84	5.84	3.18	2.60
MTORC1	1fap	0.00	0.00	0.41	0.82
OPRK1	6b73	141.67	70.83	15.00	8.33
PKM2	3gqy	4.40	4.03	2.71	2.51
PPARG	1zgy	14.81	14.81	3.70	2.96
TP53	2vuk	12.66	6.33	3.04	2.15
VDR	3a2i	0.90	2.04	1.74	1.63
**Mean**	–	**17.72**	**10.90**	**3.87**	**2.75**

Table S3: (a) Number of protein–compound pairs per protein target at different similarity thresholds between ProMol_Func training data and the LIT-PCBA library. (b) Total counts across thresholds. The methods of searching identical pairs and measuring protein_similarity × compound_similarity are described in the sections ”Removal of identical protein–small-molecule pairs” and “Protein–small-molecule similarity scoring”. For protein targets with no listed counts, this indicates that no similar protein–compound pairs were detected for that target at the corresponding similarity threshold.

(a) Per-target counts

**Protein Target**	**sim_Threshold**	**#Samples**
**Threshold 0.5**		
ADRB2	0.5	188
ALDH1	0.5	71873
ESR1_ago	0.5	60
ESR1_ant	0.5	75
FEN1	0.5	1658
GBA	0.5	9051
IDH1	0.5	23557
MAPK1	0.5	26205
OPRK1	0.5	726
PKM2	0.5	1639
PPARG	0.5	126
TP53	0.5	128
VDR	0.5	83
**Threshold 0.7**		
ALDH1	0.7	19570
ESR1_ago	0.7	7
ESR1_ant	0.7	6
FEN1	0.7	111
GBA	0.7	2475
IDH1	0.7	1698
MAPK1	0.7	7501
PKM2	0.7	212
**Threshold 0.9**		
ALDH1	0.9	8964
ESR1_ago	0.9	5
ESR1_ant	0.9	5
GBA	0.9	1011
IDH1	0.9	412
MAPK1	0.9	4510
PKM2	0.9	129
**Identical**		
IDH1	identical	209

(b) Total counts

**sim_Threshold**	**SUM**
identical	209
0.9	15036
0.7	31580
0.5	135369

Table S4: Target name, similarity threshold, and enrichment factors (EF0.5%, EF1.0%, EF5.0%, EF10.0%) based on ProMol_Func predictions for each target in the LIT-PCBA library, after removing pairs with protein_similarity*compounds_similarity >0.9 from the LIT-PCBA library. The methods of measuring protein_similarity*compounds_similarity is described in the section "Protein–small-molecule similarity scoring"

**Target**	**Threshold**	**EF_0.5%**	**EF_1.0%**	**EF_5.0%**	**EF_10.0%**
ADRB2	≤0.9	11.76	11.76	3.53	1.76
ALDH1	≤0.9	2.10	2.01	1.77	1.67
ESR1_ago	≤0.9	0.00	0.00	4.62	4.62
ESR1_ant	≤0.9	3.92	2.94	1.57	1.18
FEN1	≤0.9	24.93	17.34	5.47	3.39
GBA	≤0.9	4.91	2.45	1.96	1.72
IDH1	≤0.9	25.64	12.82	4.62	2.82
KAT2A	≤0.9	12.37	10.31	4.64	3.14
MAPK1	≤0.9	5.86	5.86	3.19	2.61
MTORC1	≤0.9	0.00	0.00	0.41	0.82
OPRK1	≤0.9	141.67	70.83	15.00	8.33
PKM2	≤0.9	4.44	3.88	2.55	2.53
PPARG	≤0.9	14.81	14.81	3.70	2.96
TP53	≤0.9	12.66	6.33	3.04	2.15
VDR	≤0.9	0.90	2.04	1.74	1.63
**Mean**	–	**17.73**	**10.89**	**3.85**	**2.76**

Table S5: Target name, similarity threshold, and enrichment factors (EF0.5%, EF1.0%, EF5.0%, EF10.0%) based on ProMol_Func predictions for each target in the LIT-PCBA library, after removing pairs with protein_similarity*compounds_similarity >0.7 from the LIT-PCBA library. The methods of measuring protein_similarity*compounds_similarity is described in the section "Protein–small-molecule similarity scoring".

**Target**	**Threshold**	**EF_0.5%**	**EF_1.0%**	**EF_5.0%**	**EF_10.0%**
ADRB2	≤0.7	11.76	11.76	3.53	1.76
ALDH1	≤0.7	2.11	2.02	1.73	1.68
ESR1_ago	≤0.7	0.00	0.00	4.62	4.62
ESR1_ant	≤0.7	3.92	2.94	1.57	1.18
FEN1	≤0.7	23.98	16.62	5.40	3.35
GBA	≤0.7	2.60	1.30	1.95	1.69
IDH1	≤0.7	25.64	12.82	4.62	2.82
KAT2A	≤0.7	12.37	10.31	4.64	3.14
MAPK1	≤0.7	6.04	5.70	3.29	2.65
MTORC1	≤0.7	0.00	0.00	0.41	0.82
OPRK1	≤0.7	141.67	70.83	15.00	8.33
PKM2	≤0.7	4.50	3.94	2.40	2.48
PPARG	≤0.7	14.81	14.81	3.70	2.96
TP53	≤0.7	12.66	6.33	3.04	2.15
VDR	≤0.7	0.90	2.04	1.74	1.63
**Mean**	–	**17.53**	**10.76**	**3.84**	**2.75**

Table S6: Target name, similarity threshold, and enrichment factors (EF0.5%, EF1.0%, EF5.0%, EF10.0%) based on ProMol_Func predictions for each target in the LIT-PCBA library, after removing pairs with protein_similarity*compounds_similarity >0.5 from the LIT-PCBA library. The methods of measuring protein_similarity*compounds_similarity is described in the section "Protein–small-molecule similarity scoring".

**Target**	**Threshold**	**EF_0.5%**	**EF_1.0%**	**EF_5.0%**	**EF_10.0%**
ADRB2	≤0.5	11.76	11.76	3.53	1.76
ALDH1	≤0.5	1.93	1.80	1.64	1.63
ESR1_ago	≤0.5	0.00	0.00	4.62	4.62
ESR1_ant	≤0.5	3.96	2.97	1.39	0.99
FEN1	≤0.5	23.08	16.48	5.27	3.30
GBA	≤0.5	0.00	0.00	1.63	1.33
IDH1	≤0.5	16.67	8.33	3.33	2.22
KAT2A	≤0.5	12.37	10.31	4.64	3.14
MAPK1	≤0.5	5.52	3.87	2.98	2.54
MTORC1	≤0.5	0.00	0.00	0.41	0.82
OPRK1	≤0.5	90.91	45.45	9.09	6.36
PKM2	≤0.5	3.98	3.59	2.19	2.31
PPARG	≤0.5	22.22	18.52	3.70	2.96
TP53	≤0.5	14.08	8.45	3.66	2.25
VDR	≤0.5	0.90	2.15	1.74	1.63
**Mean**	–	**13.83**	**8.91**	**3.32**	**2.53**

Table S7: ProMol_Func EF1% on the DUD-E test library after removing highly similar protein–compound pairs (similarity thresholds of 0.9, 0.7, and 0.5) from DUD-E. ”Sim_Threshold” refers to protein sequence identity * small molecule Morgan fingerprint Tanimoto similarity. ”Samples Removed” indicates the number of protein–compound pairs excluded for exceeding the corresponding similarity threshold.

**Sim_Threshold**	**Samples Removed**	**EF1% on DUD-E**
0.9	4125	53.92
0.7	6912	56.01
0.5	16456	56.53

Table S8: ProMol_Func EF1% on the DEKOIS2.0 test library after removing highly similar protein–compound pairs (similarity thresholds of 0.9, 0.7, and 0.5) from DEKOIS2.0. ”Sim_Threshold” refers to protein sequence identity * small molecule Morgan fingerprint Tanimoto similarity. ”Samples Removed” indicates the number of protein–compound pairs excluded for exceeding the corresponding similarity threshold.

**Sim_Threshold**	**Samples Removed**	**EF1% on DEKOIS2.0**
0.9	415	27.16
0.7	1034	30.12
0.5	1906	26.51

Table S9: ProMol_Func Precision, Recall, F1-Score and Confusion Matrix on Human HSP90 and *E. coli* DnaK(ecDnaK) test datasets across different thresholds.

Targets	Threshold	Precision	Recall	F1	Confusion Matrix
HSP90	0.4	0.822	0.845	0.833	$\begin{bmatrix} 283761 & 288 \\ 245 & 1334 \end{bmatrix}$
	0.45	0.905	0.818	0.859	$\begin{bmatrix} 283913 & 136 \\ 288 & 1291 \end{bmatrix}$
	0.5	0.941	0.788	0.858	$\begin{bmatrix} 283971 & 78 \\ 334 & 1245 \end{bmatrix}$
	0.55	0.962	0.761	0.850	$\begin{bmatrix} 284001 & 48 \\ 377 & 1202 \end{bmatrix}$
	0.6	0.973	0.707	0.819	$\begin{bmatrix} 284018 & 31 \\ 462 & 1117 \end{bmatrix}$
ecDnaK	0.4	0.286	0.333	0.308	$\begin{bmatrix} 3614 & 20 \\ 16 & 8 \end{bmatrix}$
	0.45	0.381	0.333	0.356	$\begin{bmatrix} 3621 & 13 \\ 16 & 8 \end{bmatrix}$
	0.5	0.143	0.042	0.065	$\begin{bmatrix} 3628 & 6 \\ 23 & 1 \end{bmatrix}$
	0.55	0.250	0.042	0.071	$\begin{bmatrix} 3631 & 3 \\ 23 & 1 \end{bmatrix}$
	0.6	0.333	0.042	0.074	$\begin{bmatrix} 3632 & 2 \\ 23 & 1 \end{bmatrix}$

Table S10: NanoDSF detected Tm of *E. coli* DnaK (14.1 µM), DMSO control and three known *E. coli* DnaK inhibitors. In DSF assays using extrinsic dyes (e.g., SYPRO Orange), DnaK can exhibit two apparent melting transitions that perhaps correspond to its two domains. In contrast, nanoDSF measures intrinsic Trp/Tyr fluorescence and, under our assay conditions, reports a single cooperative unfolding transition. "IP #1" refers to the first inflection point of the thermal unfolding curve. This first inflection point corresponds to the melting temperature (T_m) of the protein

**Sample ID**	**350nm IP #1 (°C)**	**330nm IP #1 (°C)**	**ΔTm (°C)**
DMSO	45.47	45.48	0.00
Quercetin (100µM)	44.83	44.82	-0.64
2-Cl-IB-MECA (100µM)	45.27	45.28	-0.20
TP (100µM)	46.74	46.76	+1.27

Table S11: NanoDSF detected Tm of *E. coli* DnaK in the presence of 100 µM EG35 or EG36 compared to DMSO control. In DSF assays using extrinsic dyes (e.g., SYPRO Orange), DnaK can exhibit two apparent melting transitions that perhaps correspond to its two domains. In contrast, nanoDSF measures intrinsic Trp/Tyr fluorescence and, under our assay conditions, reports a single cooperative unfolding transition. No significant differences in T_m were observed, indicating that there was no compound aggregation leading to protein aggregation. "IP #1" refers to the first inflection point of the thermal unfolding curve. This first inflection point corresponds to the melting temperature (T_m) of the protein

**Sample ID**	**350nm IP #1 (°C)**	**ΔTm (°C)**
DMSO (control)	43.99	0.00
EG35 (100 µM)	43.98	-0.01
EG36 (100 µM)	43.89	-0.10

Table S12: NanoDSF-measured melting temperatures (T_m) of 5 µM *M. tuberculosis* HtpG in the presence of 200 µM Geldanamycin (GA)[S1], or 100 µM EG35/EG36 were compared with the DMSO control. GA was included, as it is a well-known inhibitor of HSP90[S1]. ATP was used as a positive control. No significant changes in the T_m were observed for these three compounds, which indicated no protein aggregation. 100 µM ATP increased T_m by 5.15 °C, indicating enhanced thermal stability. GA doesn't show a Tm shift because it may not bind to this HtpG construct, as indicated by ATPase assays. "IP #1" denotes the first inflection point of the thermal unfolding curve, which corresponds to the protein's melting temperature (T_m). All values represent the mean of duplicate measurements.

**Sample ID**	**IP #1 (Ratio 350/330 nm, °C)**	**ΔT_m (°C vs DMSO)**
DMSO	42.32	0.00
GA (200 µM)	42.82	+0.50
EG35 (100 µM)	42.50	+0.18
EG36 (100 µM)	42.41	+0.09
ATP (100 µM)	47.47	+5.15

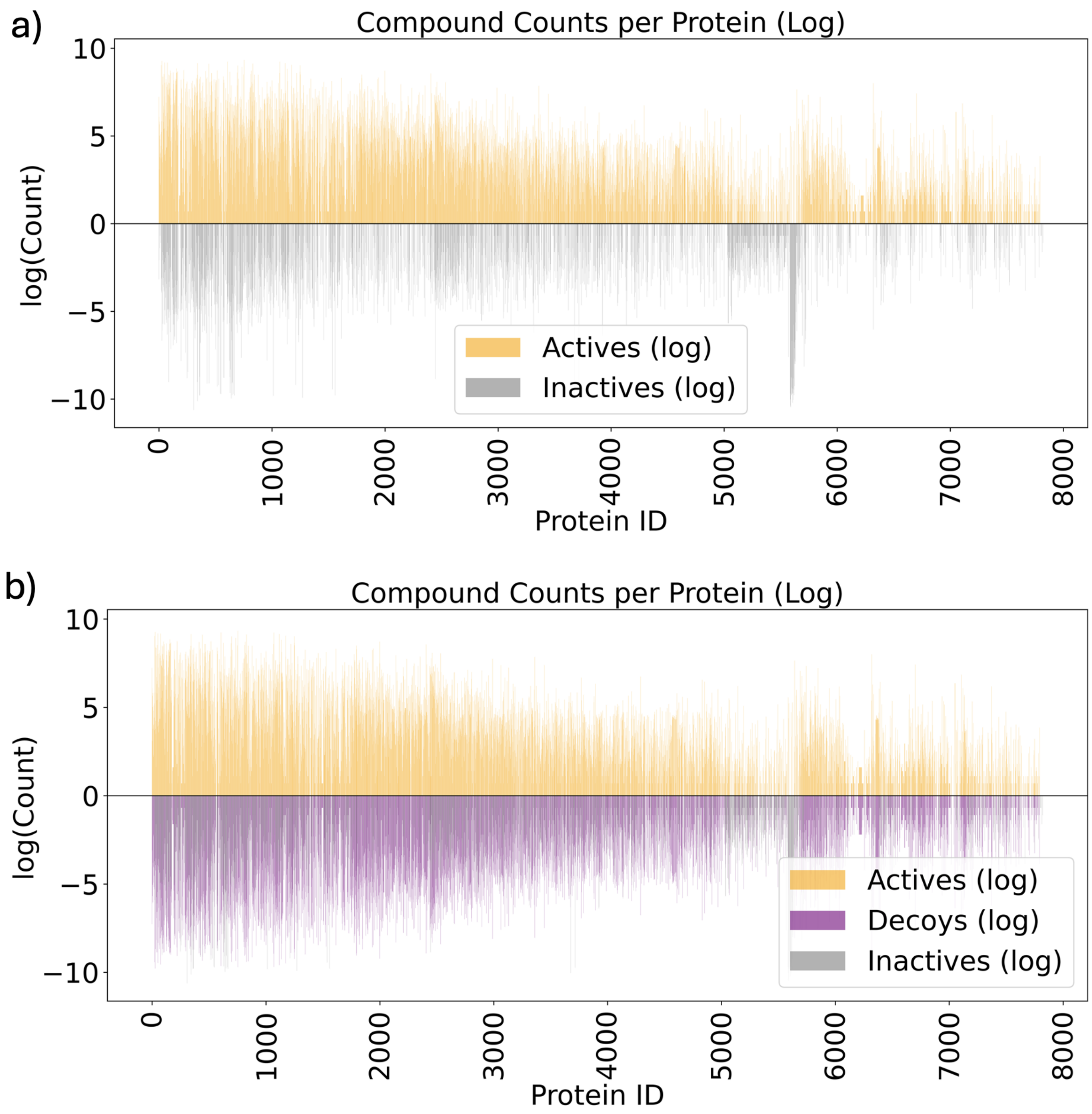

Figure S1: Log(count) distribution of: (a) experimentally validated active and inactive compounds; (b) compounds after adding randomly selected decoys for each protein index. Because many proteins in the dataset lack sufficient experimentally validated inactives, an equal number of decoys to the actives are generated and incorporated to mitigate class imbalance.

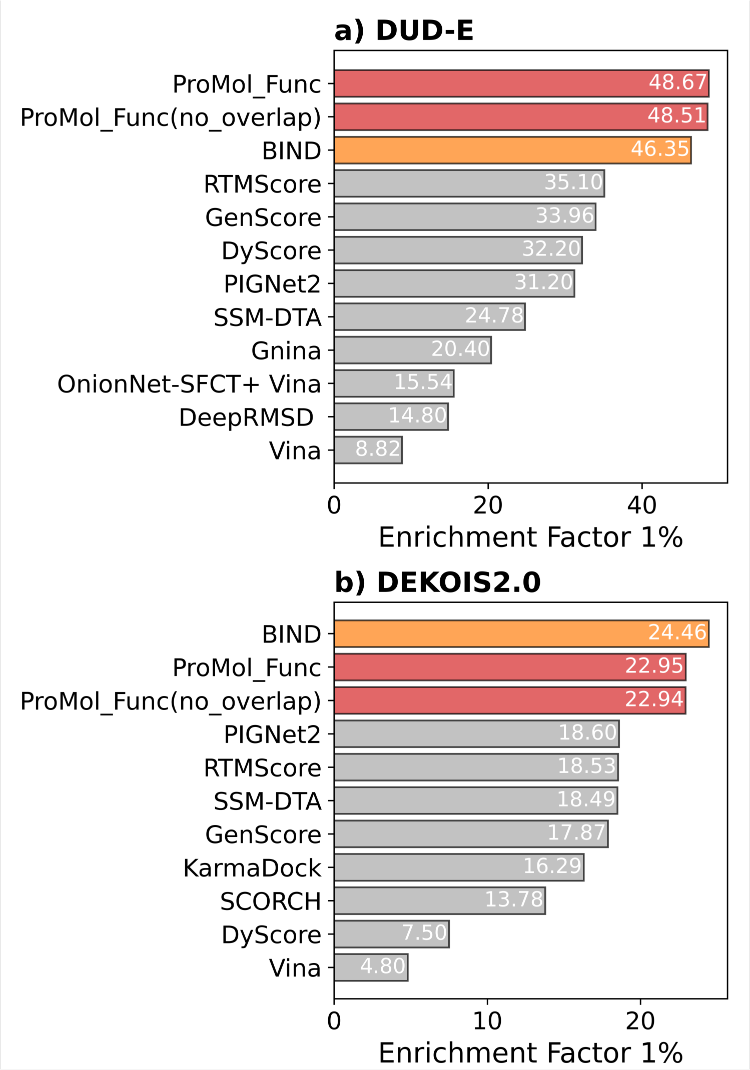

Figure S2: ProMol_Func general model(ensemble predictions of 3 models) top 1% enrichment factor (mean) on two different benchmarks. a) Performance on DUD-E dataset: 102 protein targets, 3 proteins (HIVPR, TYSY, AMPC) have overlapping actives in the training data (40 compounds for HIVPR(1xl2), 9 compounds for TYSY(1syn), and 1 compound for AMPC(1l2s)). The updated EF1% for DUD-E after removing them from test dataset is 48.51. b) Performance on DEKOIS2.0.: 81 protein targets' actives and decoys. Updated EF1% is 22.94 after removing 4 overlapped compounds for HIV1PR. All other model performance are from BIND data table[S2].

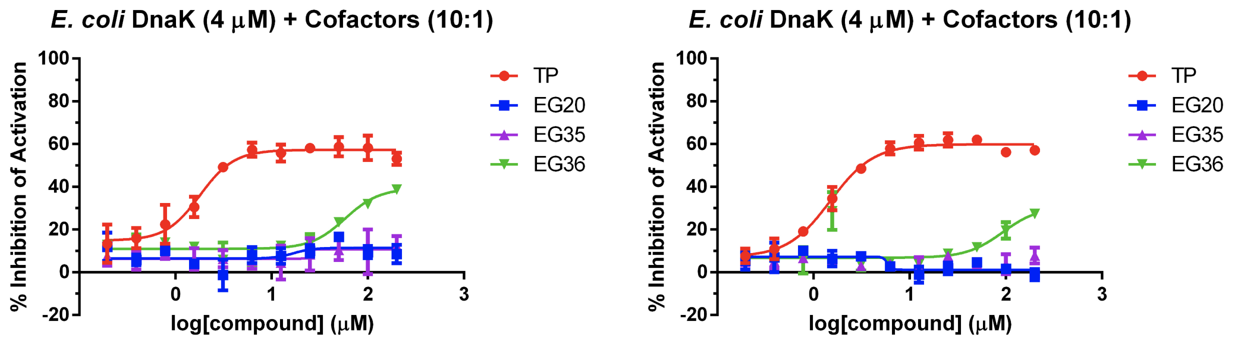

Figure S3: Two replicates of ATPase activity assay, featuring titrations of EG35 and EG36 indicate that EG36 is a better inhibitor than EG35. *E. coli* DnaK (4 μM), *E. coli* DnaJ (0.4 μM), and *E. coli* GrpE (0.4 μM). To calculate "Inhibition of Activation", reactions containing DnaK, DnaJ, GrpE, and compound were normalized to control reactions containing *E. coli* DnaK with 10% DMSO (100% inhibition of activation) and reactions containing *E. coli* DnaK-cofactors with 10% DMSO (0% inhibition of activation). For each experiment n=3, error bars represent standard deviation (SD).

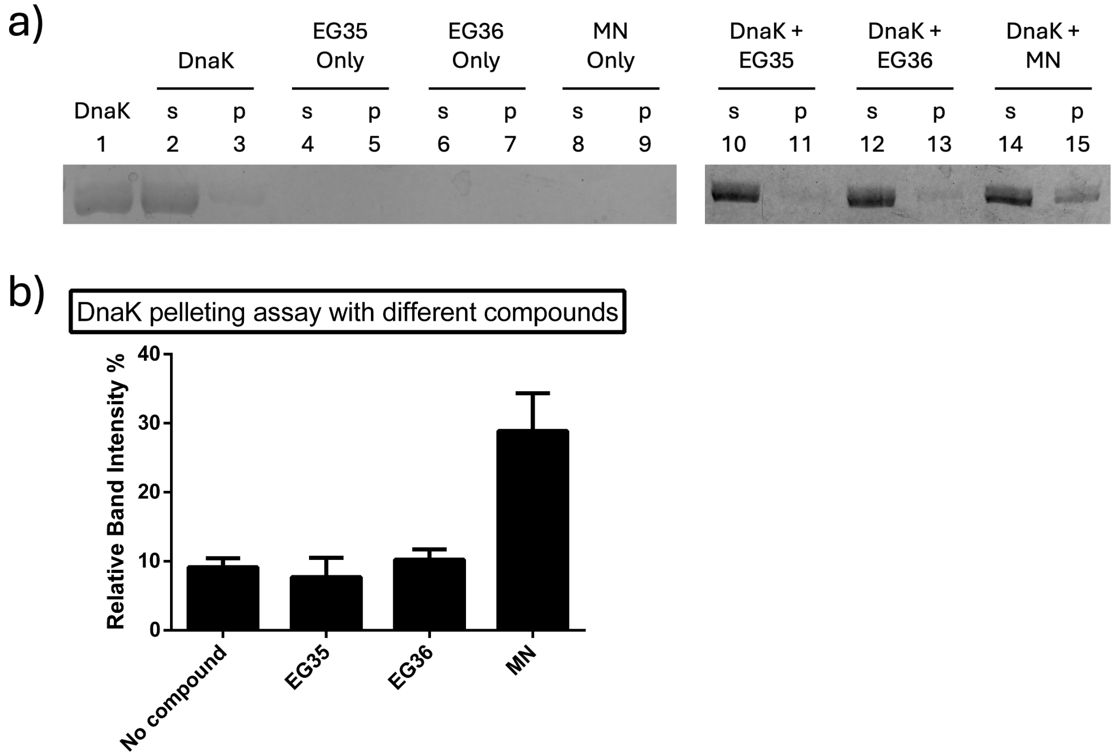

Figure S4: EG35 and EG36 do not aggregate protein under ATPase assay reaction conditions. (a) Representative SDS-PAGE analysis of supernatant (s) and pellet (p) following centrifugation (15493 x g, 30 min, 25ºC) of 4 µM *E. coli* DnaK with or without 200 µM compound in ATPase assay buffer (50 mM HEPES, pH 7.5, 2 mM $MgCl_2$, 0.01% Tween-20, 20% DMSO). Lanes 1-3 represent DnaK without compound. Lanes 4-9 represent compound only. Lanes 10-15 represent combined DnaK with indicated compounds. Miconazole nitrate (MN) is a promiscuous aggregate-forming compound[S3]. (b) Relative band intensity was calculated as the ratio of the pellet intensity relative to the pellet and supernatant intensities combined[S4] (n = 3, bars represent SD). The lower ratios of EG35 and EG36 compounds in the pellet compared to MN suggest they do not co-sediment with DnaK after centrifugation and therefore do not aggregate protein under ATPase assay reaction conditions.

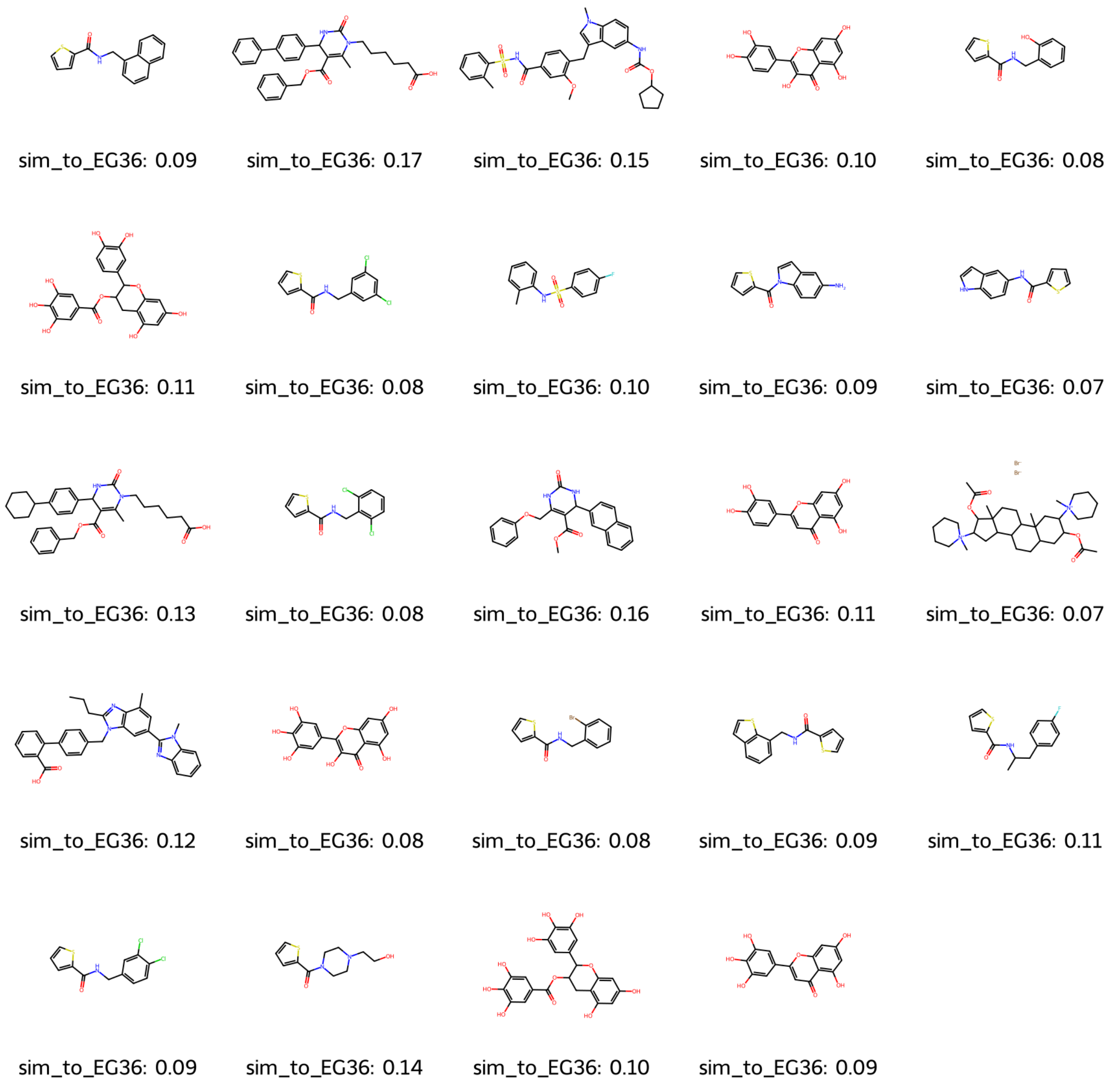

Figure S5: Tanimoto similarity of Morgan fingerprints (radius = 2) between ProMol_Func identified inhibitor EG36 and 24 collected *E. coli* DnaK inhibitors in the test set.

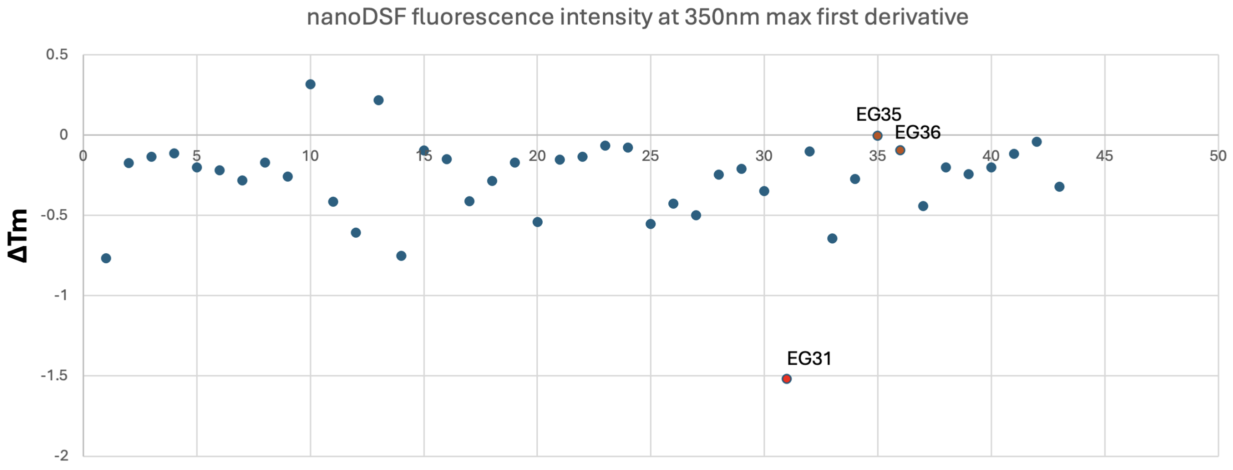

Figure S6: Nano differential scanning fluorimetry (nanoDSF) analysis of 43 compounds predicted by ProMol_Func to bind *E. coli* DnaK via zero shot learning. Concentration ranges from 100 µM to 1 mM depending on their solubility in DMSO. 2 compounds showed an increased Tm of *E. coli* DnaK (14.1 µM), and 8 compounds showed a decreased Tm ($> 0.5\,°C$ change) of the same concentration of DnaK compared to the DMSO control. 500 µM EG31 showed a decreased Tm of *E. coli* DnaK (14.1 µM) of $\sim 1.5\,°C$ compared to the DMSO control. Neither 100 µM EG35 nor 100 µM EG36 produced a significant change in the Tm of *E. coli* DnaK (14.1 µM).

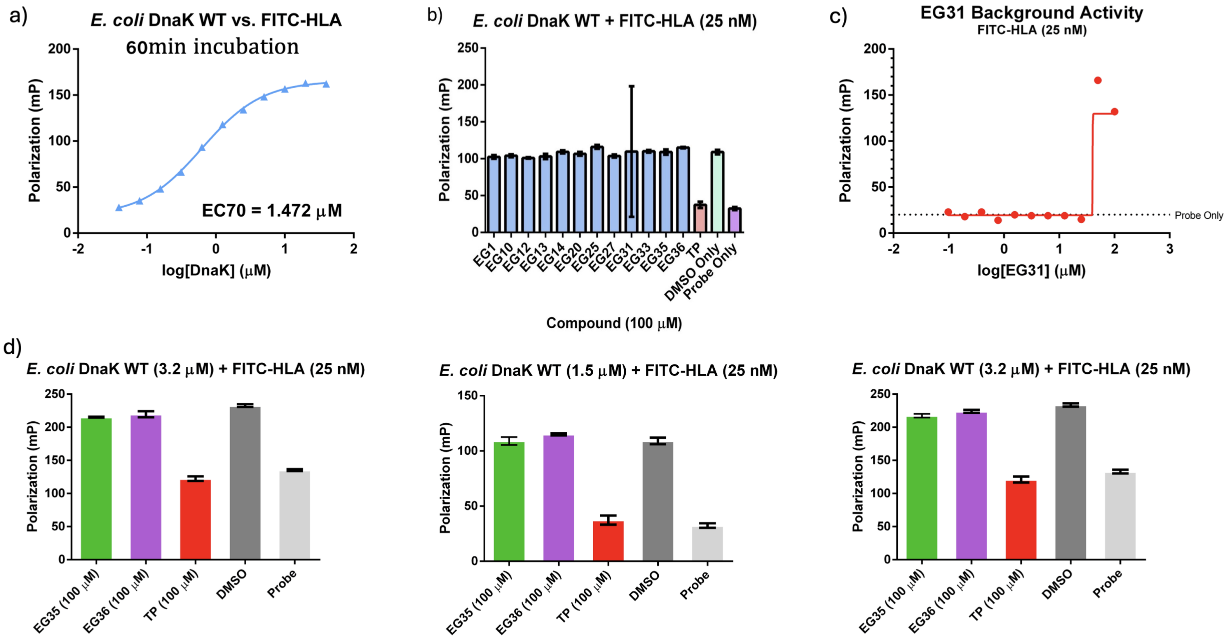

Figure S7: EG compounds do not bind to the SBD peptide-binding pocket of DnaK. a)Fluorescence Polarization (FP) saturation binding assays using a published fluorophore-labeled probe FITC-HLA (FITC-$\beta A \beta A$-RENLRIALRY).[S5–S7] b)FP-based displacement assay using a fluorescently labeled peptide ligand (FITC-HLA) indicates the EG compounds do not bind to the peptide binding cleft, unlike telaprevir (TP) (100 µM EG35, EG36, and TP (positive control)). c) FP-based analysis of increasing [EG31] with probe alone indicates interaction of EG31 with probe at high concentrations, which likely led to high error in part b. d) Three replicates of FP-based displacement assay for EG35, EG36, and TP using FITC-HLA. Consistently, only TP displaced the probe. ”Probe” in the graph means FITC-HLA control without protein added. For each experiment n=3, error bards represent standard deviation (SD).

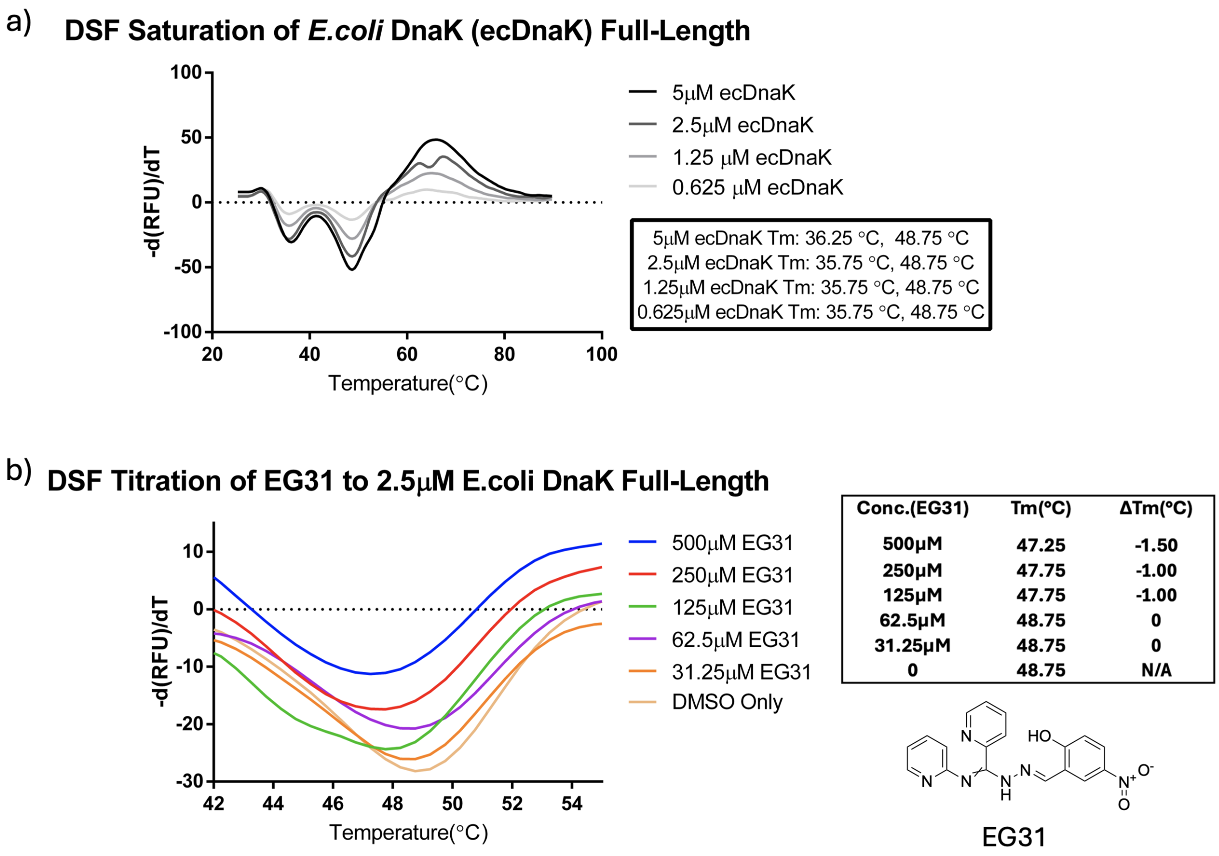

Figure S8: Differential Scanning Fluorimetry (DSF) analysis with SYPRO dye of DnaK with select compounds. (a) Different concentration curve of *E. coli* DnaK (duplicates). There were two detected Tm values of *E. coli* DnaK, perhaps due to melting of two domains or different oligomer states[S8,S9]; b) Titration of EG31 to 2.5 µM *E. coli* DnaK. Temperature was increased from 25°C to 90°C with an increment at 0.50°C per cycle. Y=-$\Delta RFU/\Delta Temperature$. Tm is the temperature where the maximum absolute number of negative first derivative happens. Chemical structure of EG31 is at right.

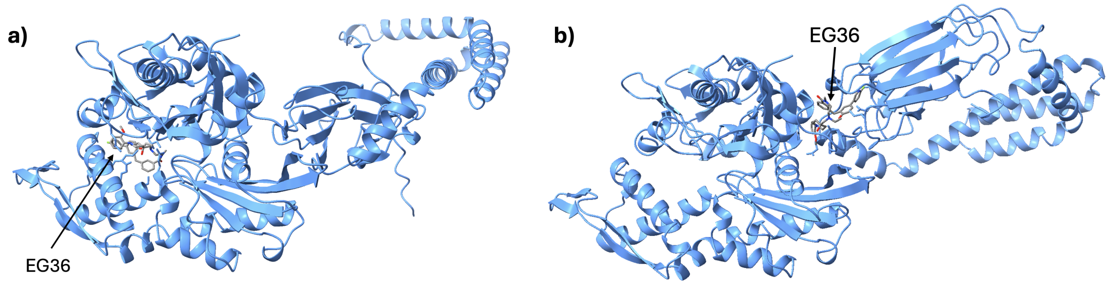

Figure S9: a) Alphafold3[S10] predicted pose with the highest-ranking score of EG36 bound to an *E. coli* DnaK NBD allosteric site. b) Boltz-2[S11] predicted that EG36 binds to the linker domain between NBD and SBD

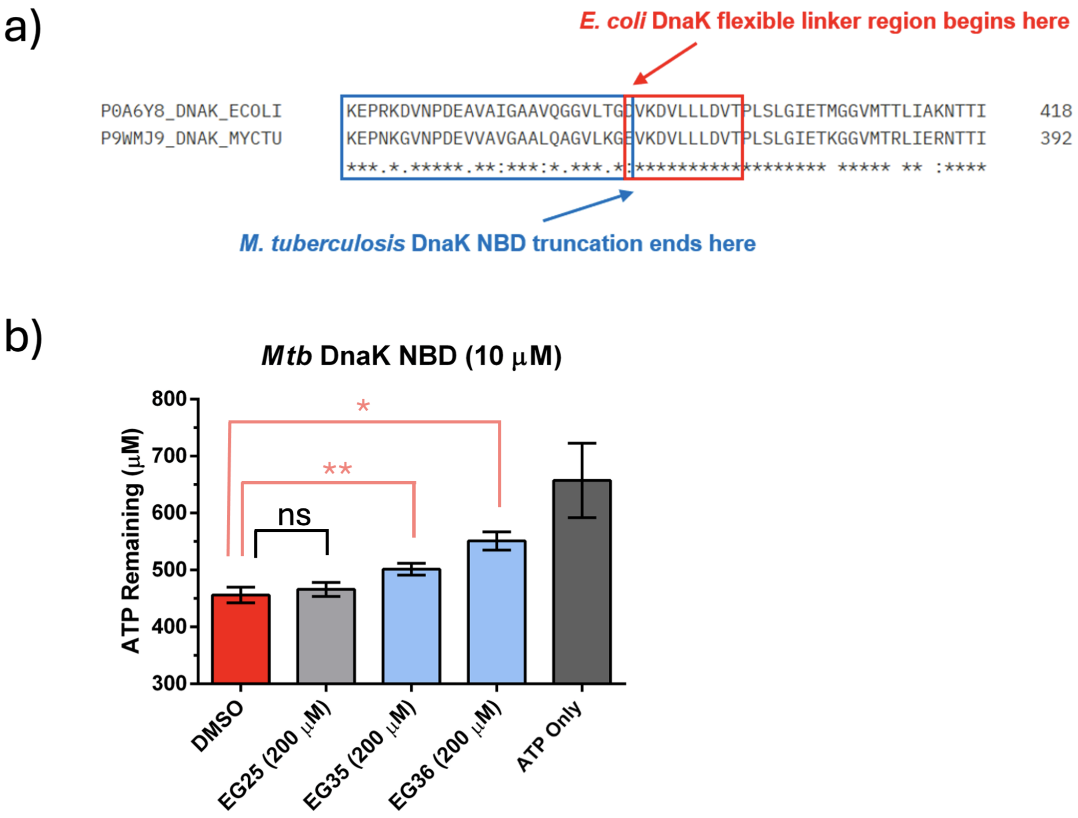

Figure S10: Compound effects on ATPase activity of *M. tuberculosis* (*M. tb*) DnaK nucleotide-binding domain (NBD; residues M1–E359). a) Sequence alignment of *E. coli* DnaK and *M. tb* DnaK NBD to verify that our NBD truncation lacks the flexible linker. b) ATPase activity of the *M. tb* DnaK NBD in the presence of indicated EG compounds ($n = 3$; bars represent SD). EG35 and EG36 significantly reduced ATPase activity relative to DMSO, while EG25, an inactive compound, showed no significant effect. *E. coli* and *M. tb* DnaK share 56.6% sequence identity, supporting that EG35 and EG36 function as broad-spectrum bacterial DnaK inhibitors, rather than being restricted to *E. coli*-specific inhibition. *M. tb* DnaK was also used because it represents a possible drug target to for anti-tuberculosis strategies[S7]. Note, this graph shows ATP remaining. ($^{*}p < 0.05$, $^{**}p < 0.0025$; ns = not significant. A two-tailed paired t-test was used for comparisons.)

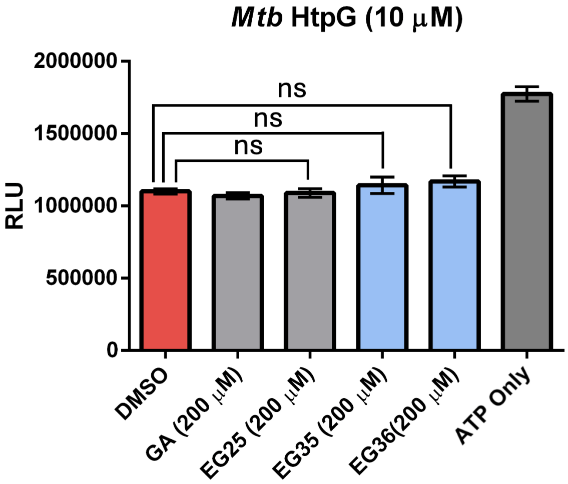

Figure S11: Compound effects on ATPase activity of *M. tb* HtpG (n = 3, bars represent SD). *M. tb* HtpG (10 μM) was incubated with or without compounds (200 μM) at 30°C. Geldanamycin (GA) was active against some HtpG constructs[S12], but was not active under the conditions tested. Raw luminescence values (RLU) directly correspond to the amount of ATP left in solution via Kinase-Glo Max detection (Promega). No significant (ns) differences were observed between DMSO and EG25, EG35, or EG36. Therefore, EG35 and EG36 do not inhibit HtpG ATPase activity. EG25 is an inactive compound included for comparison. ($P > 0.05$ for all comparisons, ns=not significant. A two-tailed paired t-test was used for comparison)

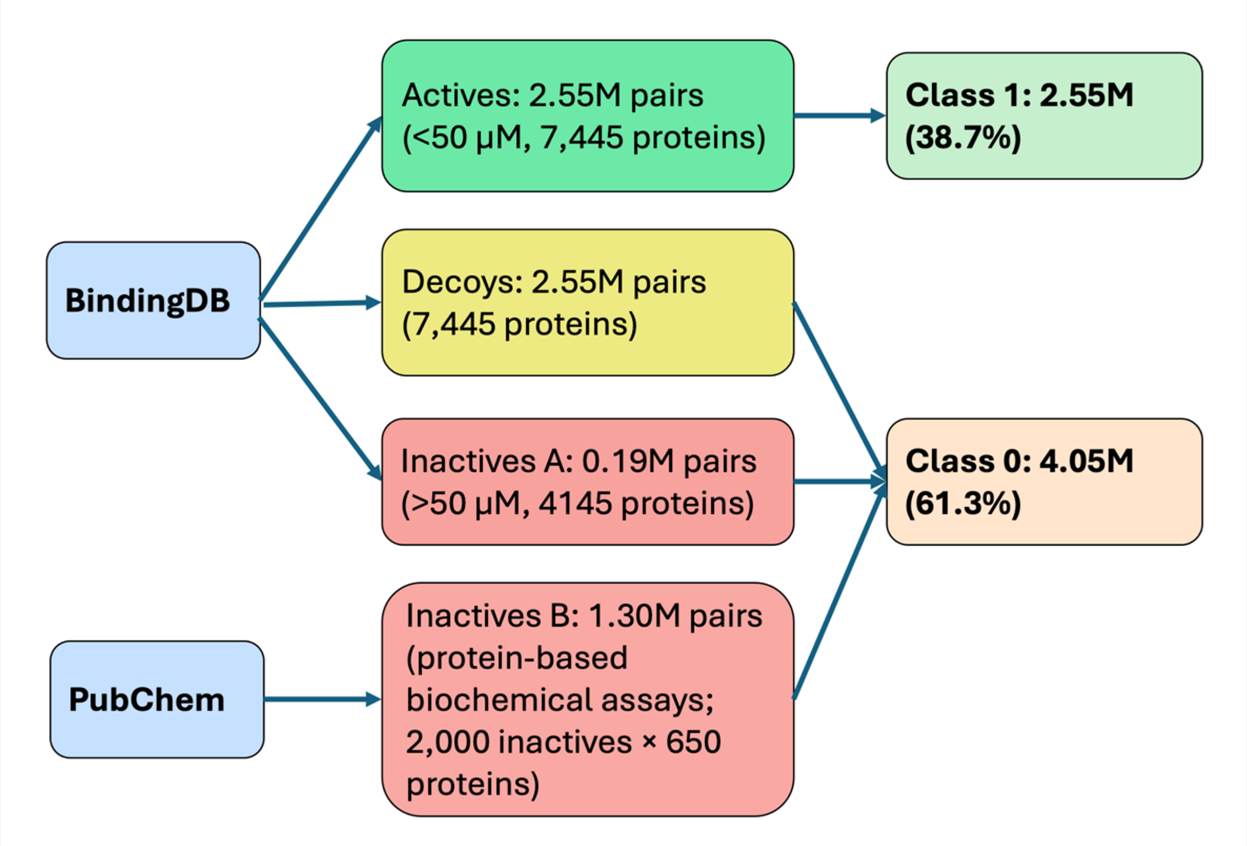

Scheme S1. Scheme of ProMol_Func Dataset construction. A total of 2.55 million protein–ligand pairs across 7,445 proteins with any reported metric (K_I, IC_{50}, EC_{50}, or K_D) below 50 µM in BindingDB were designated as positives (Class = 1). Inactives consisted of 0.19 million BindingDB protein–ligand pairs with activity values above 50 µM, together with 1.30 million additional inactives randomly sampled from PubChem (2,000 inactives per protein for 650 proteins). In addition, 2.55 million decoys were generated from BindingDB by randomly selecting ligands of other proteins that do not correspond to the target protein. Inactives and decoys were designated as negatives (Class = 0). The curated dataset was scaffold-balanced split into training, validation, and test sets at an 8:1:1 ratio.

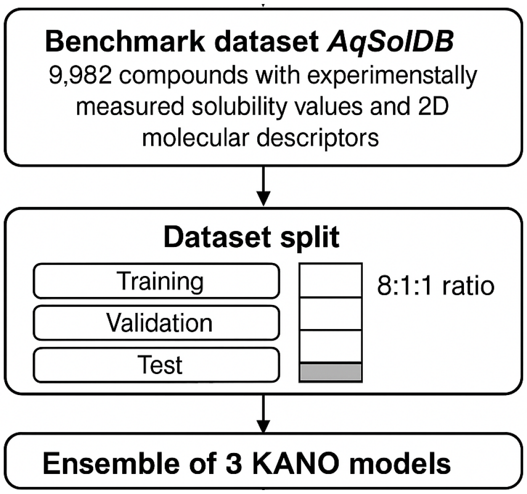

Scheme S2. Fine-tuning of the KANO model for aqueous solubility prediction using the benchmark dataset AqSolDB, which consists of 9,982 compounds with experimentally measured solubility values and associated 2D molecular descriptors[S13]. The dataset was randomly split into training, validation, and test sets in an 8:1:1 ratio. Model performance was evaluated by using an ensemble of three independently trained KANO models[S14], and final predictions were obtained by averaging the ensemble outputs. This scheme was generated with the assistance of ChatGPT (OpenAI).

Supporting Computational Methods

BindingDB Pilot Subset Used to Compare Protein–Ligand Embedding Combinations. To compare addition versus concatenation of DeepFRI protein-function embeddings with KANO embeddings, we first constructed a pilot dataset from BindingDB. This dataset comprised 0.247M protein–ligand pairs designated as actives (with inhibition constant half-maximal inhibitory concentration (IC_{50}) $<$ 10 nM) and 0.187 M pairs designated as inactives (with IC_{50} $>$ 25 µM). The pilot dataset was scaffold-balanced and split into training, validation, and test sets in an 8:1:1 ratio. Performance comparisons between addition and concatenation are summarized in Figure S1. In contrast, subsequent training of ProMol_Func employed a substantially larger BindingDB subset constructed with a universal 50 µM activity threshold.

ProMol_Func Dataset Curation. The data construction is shown in Scheme S1. 2.55M active and 0.19M inactive Protein-ligand pairs were from BindingDB with available metrics including inhibition constant (K_I), half-maximal inhibitory concentration (IC_{50}),

half-maximal effective Concentration (EC_{50}), or dissociation constant (K_D). The threshold for inactives from BindingDB is the protein ligand with any of the metrics larger than 50 µM, otherwise, it is considered as actives. This 50 µM cutoff was chosen as a practical, screening-consistent threshold for large-scale model training across 7817 protein targets. A critical literature survey of more than 400 virtual screening campaigns by Zhu et al.[S15] showed that most studies used activity cutoffs in the low- to mid-micromolar range, with many campaigns adopting thresholds between 1–25 µM and 25–50 µM and a substantial number using 50–100 µM or even 100–500 µM (or higher) as the initial cutoff. They also highlighted that relatively weak but structurally novel hits are often deliberately accepted to maximize chemical diversity and to enable discovery against novel targets with limited prior inhibitor knowledge.[S15] In parallel, the LIT-PCBA benchmark reported by Tran-Nguyen et al.[S16] is constructed from high-confidence dose–response PubChem bioassays and exhibits a potency distribution for actives that is predominantly micromolar (median pActivity ≈ 5.2), reflecting affinities typically observed in HTS campaigns.[S16] Within this context, a 50 µM threshold provides a transparent and reproducible operational definition of "active" that is compatible with common virtual screening practice and with the potency regime represented in LIT-PCBA, while enabling consistent treatment of heterogeneous assay data at scale. We emphasize that this cutoff is intended as a practical choice for training a ranking model to prioritize likely binders, rather than as a claim that 50 µM represents an optimal or universal pharmacological boundary for every individual target.

In addition, we incorporated bioassay data from PubChem. Out of the nearly two million bioassays in PubChem, 1,494 include annotated protein targets with URL links to PubChem protein target pages. Among these, some are protein-based assays including binding and functional assays, while others are cell-based assays involving those proteins. From these, we specifically downloaded data of protein-based assays and extracted their inactive compounds (compounds annotated as "inactive"). To balance the dataset, we randomly sampled 2,000 inactive compounds per protein from PubChem biochemical assays (including both primary

and confirmatory assays) for proteins not represented in the LIT-PCBA library, yielding 650 protein targets. These randomly sampled inactives were then combined with the compounds collected from BindingDB. The protein sequences were obtained based on the related NCBI entry extracted from their Target URL in Pubchem website.

Because many proteins lack sufficient numbers of experimentally validated inactive compounds, we randomly sampled decoys from BindingDB to supplement the negative class (Figure S1). In this study, the term "decoys" refers to assumed negatives,consistent with established virtual screening benchmarks such as DUD-E[S17] and DEKOIS2.0[S18] and recent large-scale studies[S2,S19]. In our work, decoys are employed as a data enrichment strategy to address class imbalance. These decoys were collected exclusively from BindingDB and consist of compounds that are experimentally confirmed binders to other protein targets but have no reported binding activity toward a given protein. These compounds are treated as assumed negatives (class = 0) for that protein, and analogously for other proteins. For each protein, decoys were randomly sampled to match the number of active compounds, ensuring that each protein had a sufficient and balanced set of negative samples. We acknowledge that this may introduce a limited degree of noise and potential false negatives. However, both assumed negatives (decoys) and labeled inactives therefore play complementary roles in mitigating class imbalance and enriching the negative space, as illustrated in Figure S1. Our primary objective is to train a ranking model that learns to prioritize true binders in a large, diverse chemical and protein space, rather than to construct a perfectly noise-free classification dataset. Finally, there are 7817 proteins and their 2.55M actives, 2.55M decoys, and 1.49M inactives (6.60M pairs in total). However, some SMILES strings were invalid and were excluded from the training process.

ProMol_Func general model Evaluation Datasets. DEKOIS 2.0[S18], DUD-E[S17] and LIT-PCBA[S16] datasets were used to determine screening power. For all datasets, protein sequences were converted from pdb files using pdb2fasta. In instances where multiple sequences relating to the target were present, the main chain sequence of monomer binding to

the ligand was saved. Top 0.5%, 1%, 5% and 10% enrichment were calculated and top1% EF was compared to other models. Sequences for protein targets from LIT-PCBA were retrived based on pdb IDs in Table S2. All SMILES need to be utilized to canonical SMILES to check validity.

General Model Training

The addition of protein function to small molecules graph encoders during model training outperformed concatenation, demonstrating higher accuracy on both the validation and test sets split from a pilot dataset, as shown in Table S1. Furthermore, the addition approach resulted in a model with fewer parameters, offering a more efficient solution without compromising performance (Table S1). Based on DeepFRI's molecular functions (MF) predictions, a linear layer that inputs 489 features to output 300 features was used to embed protein functions. Then, the protein embeddings with a [1, 300] vector size were added to the CMPN-generated small molecule representations, which also have a size of [1, 300]. 5 layers of feed forward network were then applied. The ProMol_Func Dataset was scaffold-balanced split with ratio at 8:1:1 for training/validation/test. Three models were then trained using different random seeds for scaffold-balanced splitting (seeds=1, 2, and 3), and their predictions were combined through ensemble averaging. Metric for validation is accuracy. ProMol_Func was finished training for 10 epochs over 3 to 4 days using a single Nvidia A100 GPU, with an initial learning rate at 0.0001 and a batch size of 256. The model has 2,596,706 parameters in total. Best validation model – models with best validation performance – were saved for test evaluation.

Removal of identical protein–small-molecule pairs

A protein–small-molecule pair is considered identical to another if they share the same protein amino acid sequence and the same small molecule (as determined by canonical SMILES). Such duplicate pairs are removed from test sets to ensure that no exactly repeated entries

are shared between datasets.

Protein–small-molecule similarity scoring

We score a protein–small-molecule pair (x, s) from the test set against a protein–small-molecules pair (y, t) from the training set by multiplying (i) the global pairwise alignment identity between protein sequences x and y[S20] and (ii) the Morgan fingerprint Tanimoto similarity between ligands s and t[S21,S22].

Protein sequence identity. Sequences x and y are globally aligned[S20]. Let $\tilde{x}, \tilde{y}$ denote the aligned sequences of equal length L (including gaps). The percent identity is computed as the fraction of aligned positions with identical characters:

$$\mathrm{Id}(x, y) \;=\; \frac{1}{L} \sum_{k=1}^{L} \mathbf{1}[\tilde{x}_k = \tilde{y}_k]\,, \tag{1}$$

where $\mathbf{1}[\cdot]$ is the indicator function. In our implementation, the alignment scoring uses match $= 1.0$, mismatch $= 0.0$, gap-open $= -1.0$, and gap-extend $= -0.5$.

Small molecules similarity (Morgan–Tanimoto). Each SMILES string is transformed into a binary Morgan fingerprint (ECFP) with radius $r = 2$ and 2048 bits. Let $f(s), f(t) \in \{0, 1\}^{2048}$ be the fingerprints for small molecules s and t. Define

$$a = \|f(s)\|_1, \quad b = \|f(t)\|_1, \quad c = \|f(s) \wedge f(t)\|_1,$$

where $\wedge$ is bitwise AND and $\|\cdot\|_1$ counts set bits. The Tanimoto similarity is

$$\mathrm{Tan}(s, t) \;=\; \frac{c}{a + b - c}. \tag{2}$$

Combined score and decision rule. The overall similarity is the product

$$S\Big((x,s),(y,t)\Big) \;=\; \mathrm{Id}(x,y) \;\times\; \mathrm{Tan}(s,t). \tag{3}$$

We remove a pair from test datasets (LIT-PCBA, DUD-E, DEKOIS2.0) when the combined score exceeds a chosen threshold τ. We use three stringency levels $\tau \in \{0.9,\, 0.7,\, 0.5\}$ (strict, medium, lenient).

$$\text{remove if} \quad S\Big((x,s),(y,t)\Big) \;\geq\; \tau, \qquad \tau \in \{0.9,\, 0.7,\, 0.5\}. \tag{4}$$

All reported results of removing "similar" protein-ligand pairs from test datasets in this work use the protein-ligand similarity scoring in Eqs. (1)–(4).

Model performance evaluation metric

The Enrichment Factor (EF) is a metric used to evaluate the ability of a ranking model to identify true binders among the top-ranked candidates. It measures how much better a model performs in identifying true binders compared to random selection:

$$\mathrm{EF}_\alpha = \frac{NTB_\alpha}{NTB_{\mathrm{total}} \cdot \alpha}$$

NTB_α is the number of true binders observed among the top-ranked candidates (e.g., $\alpha = 1\%$, 5%, or 10%), $\mathrm{NTB}_{\mathrm{total}}$ is the number of true binders in total. Precision measures the proportion of correctly predicted positive samples out of all samples predicted as positive. It indicates how many of the predicted binders are actually true binders:

$$\mathrm{Precision} = \frac{\mathrm{TP}}{\mathrm{TP} + \mathrm{FP}}$$

Recall measures the proportion of actual positive samples that were correctly predicted as

positive. It indicates how many of the actual binders were identified by the model:

$$\text{Recall} = \frac{\text{TP}}{\text{TP} + \text{FN}}$$

The F1-score is the harmonic mean of precision and recall. It provides a balanced measure when there is an uneven class distribution. A high F1-score indicates a good balance between precision and recall:

$$F1\text{-score} = 2 \times \frac{\text{Precision} \times \text{Recall}}{\text{Precision} + \text{Recall}}$$

Supporting Experimental Methods

Expression and Purification of Bacterial Chaperones

Escherichia coli chaperones/cofactors (DnaK, DnaJ, and GrpE) were expressed and purified by a similar method to that described previously (Hosfelt et al., 2022)[S7], with slight modifications described below. *E. coli* DnaK, DnaJ, and GrpE were overexpressed in *E. coli* Rosetta2 pLysS cells. Overnight cultures in 5 mL of LB (carbenicillin 50 µg/mL, chloramphenicol 30 µg/mL, 1% glucose (v/v)) were inoculated into 500 mL LB containing carbenicillin 50 µg/mL, chloramphenicol 30 µg/mL, and 0.04% glucose (v/v), and were grown with shaking (37°C, 200 rpm) to an OD600 (optical density at 600 nm) of 0.4 to 0.6, following induction for 3 to 4 hours with 1 mM IPTG (isopropyl -D-thiogalactopyranoside) at 25°C.

Each DnaK and GrpE cell pellet (from 500 mL culture) was resuspended in Buffer A (25 mM Tris-(hydroxymethyl)aminomethane (Tris), pH 8.0, 400 mM NaCl, 10% glycerol (v/v)) with lysozyme (0.1 mg/mL) and DNaseI (6.6 ug/mL). The resuspended pellet was rocked at 4°C for 1 hour and lysed by high-pressure homogenization. Cellular debris was removed from the lysate by ultracentrifugation (Beckman Coulter Allegra X-15R, 10956 x g, 40 min, 4°C). The remaining supernatant was loaded onto a pre-washed equilibrated 20 mL Ni-NTA column (Qiagen) containing 2 mM imidazole and rocked at 4°C for 1 hour.

Protein bound to the column was washed with 8 column volumes (CV) of 30 mM imidazole in Buffer A (25 mM Tris-(hydroxymethyl)aminomethane (Tris), pH 8.0, 400 mM NaCl, 10% glycerol (v/v)) with lysozyme (0.1 mg/mL) and DNaseI (6.6 ug/mL) and eluted with 1 CV of 200 mM, 300 mM, and 500 mM imidazole in Buffer A. Eluted protein was combined with SUMO protease His-Ulp1 (2100 ug) and dialyzed overnight using Snakeskin Dialysis Tubing (Thermo Scientific, 10K MWCO, 35 mm) in 2L Buffer A at 4°C. The dialyzed solution was loaded into a pre-washed equilibrated 20 mL Ni-NTA column (Qiagen) and rocked at 4°C for 1 hour. The protein sample was collected (called 'flow-through') and the column was washed with 1 CV Buffer A and 10 mM imidazole in Buffer A. Flow-through and column washes were concentrated to 1 mL and buffer exchanged into Buffer B (50 mM HEPES, 50 mM NaCl, pH 7.5) using an Amicon centrifugal unit (MilliporeSigma, 10K MWCO, 1857 x g, 4°C). Precipitated protein was removed by centrifugation (Beckman Coulter Microfuge 20R, 10,000 x g, 2 min, 4°C) and was further purified via anion exchange using a 1 mL HiTrap Q HP column (Cytiva) in Buffer B and an increasing gradient of Buffer D (50 mM HEPES, 1 M NaCl, pH 7.5). Fractions from the first clean peak were pooled together, concentrated, and buffer exchanged into Buffer A, and further concentrated to 1 mL using an Amicon centrifugal unit (MilliporeSigma, 10K MWCO, 1857 x g, 4°C). The precipitated protein was removed by centrifugation (Beckman Coulter Microfuge 20R, 10,000 x g, 2 min, 4°C).

Each DnaJ cell pellet (from 500 mL culture) was resuspended in Buffer A (25 mM Tris(hydroxymethyl)aminomethane (Tris), pH 8.0, 400 mM NaCl, 10% glycerol (v/v)) with lysozyme (0.1 mg/mL) and DNaseI (6.6 ug/mL), and 1X HaltTM Protease Inhibitor Cocktail (Thermo Scientific). The resuspended pellet was rocked at 4°C for 1 hour and lysed by sonication (5 min, 50% amplitude, 30 sec on/off) for three cycles. Cellular debris was removed from the lysate by ultracentrifugation (Beckman Coulter Allegra X-15R, 10956 x g, 30 min, 4°C). The remaining supernatant was loaded into a pre-washed equilibrated 5 mL Ni-NTA column (Qiagen) containing 2 mM imidazole and rocked at 4°C for 1 hour. Protein bound to the column was washed with 5 column volumes (CV) of 30 mM imidazole

in Buffer A and eluted with 1 CV of 200 mM, 300 mM, and 500 mM imidazole in Buffer A. Eluted protein was combined with SUMO protease His-Ulp1 (1400 ug) and dialyzed overnight using Snakeskin Dialysis Tubing (Thermo Scientific, 10K MWCO, 35 mm) in 2L Buffer A at 4°C. The dialyzed solution was loaded into a pre-washed equilibrated 10 mL Ni-NTA washed resin (Qiagen) and rocked at 4°C for 30 minutes. The protein sample was collected (called 'flow-through'), and the column was washed with 1 CV of Buffer A and a gradient of increasing imidazole (10 mM, 20 mM, 30 mM, 50 mM, 100 mM, and 200 mM) in Buffer A. The flow-through and 30 to 100 mM imidazole washes were combined and concentrated to 6 mL using an Amicon centrifugal unit (MilliporeSigma, 10K MWCO, 1857 x g, 4°C). Protein was dialyzed overnight using Snakeskin Dialysis Tubing (Thermo Scientific, 10K MWCO, 35 mm) in 2L Buffer A at 4°C to remove imidazole. Protein samples were then concentrated to 1 mL and buffer exchanged into Buffer C (25 mM Tris, pH 8.0, 50 mM NaCl, 10% glycerol) using Amicon centrifugal unit (MilliporeSigma, 10K MWCO, 1857 x g, 4°C). Precipitated DnaJ was removed by centrifugation (Beckman Coulter Microfuge 20R, 10,000 x g, 2 min, 4°C) and was further purified via anion exchange using a 1 mL HiTrap Q HP column (Cytiva) in Buffer C and an increasing gradient of Buffer D (25 mM Tris, pH 8.0, 1 M NaCl, 10% glycerol). The fractions from the first clean peak were pooled together, concentrated, and buffer exchanged into Buffer A, and further concentrated to 500 µL using an Amicon centrifugal unit (MilliporeSigma, 10K MWCO, 1857 x g, 4°C). The precipitated protein was removed by centrifugation (Beckman Coulter Microfuge 20R, 10,000 x g, 2 min, 4°C) and purified further on the Superdex 200 increase 10/30 GL column with Buffer A. Fractions were analyzed using an SDS-PAGE gel (4-20%) and clean fractions were combined and concentrated using an Amicon centrifugal unit (MilliporeSigma, 10K MWCO, 1857 x g, 4°C).

All purification steps were performed at 4°C or on ice. The protein concentration was estimated by DC Protein Assay (Bio-Rad), using BSA (bovine serum albumin) as the standard. Following the purification, all proteins were frozen in N2(l) and stored at -80°C until

use.

Screen of Inhibitors of *E. coli* DnaK-cofactor ATPase activity

This protocol was adapted from those previously described (Chang et al., 2008)[S23]. Initial screen reactions were performed at 30 µL final volume in a 96-well flat-bottom polystyrene clear microplate (Greiner). The microplate was spun down in a Sorvall ST16 (ThermoFisher Scientific) centrifuge for 1 minute at 142 x g after the addition of each reaction component except the malachite green reagent. The final reaction mixture consisted of *E. coli* DnaK (4 µM), DnaJ (0.4 µM), and GrpE (0.4 µM) were diluted in reaction buffer containing Buffer B (50 mM HEPES pH 7.5, 2 mM $MgCl_2$, and 0.01% Tween-20). To achieve a final compound concentration of 100 µM in 10% (v/v) DMSO, 3 µL of compound (1 mM in DMSO) was added to each reaction. DnaK and compound were added to the wells first and incubated at 37°C for 20 minutes before adding DnaJ and GrpE. After another incubation at 37°C for 20 minutes, reactions were initiated by adding 3 µL of 1 mM ATP to reach a final ATP concentration of 100 M. The plate was left to incubate at 37°C for 30 minutes before each reaction was quenched with 30 µL of 0.05% formic acid. Malachite green (MG) detection reagent was created by combining 0.0812% (w/v) malachite green oxalate in H2O, 2.32% (w/v) polyvinyl alcohol in H2O, 5.72% (w/v) ammonium molybdate in 6N HCl, and H2O in a 2:1:1:2 ratio, respectively. MG reagent was incubated at room temperature for 10 minutes before adding 60 µL to each quenched reaction. The plate was then left to incubate at room temperature for 30 minutes. Absorbance at 630 nm was measured using an Agilent Biotek Synergy H1 plate reader. The absorbance was converted to the concentration of phosphate produced in each reaction using a standard curve generated with potassium phosphate in an identical buffer.

Compound titration using *E. coli* DnaK-mediated ATP Hydrolysis (ATPase) Assays

Compound dose-response experiments were adapted from previously reported protocols (Richards et al., 2022)[S24], but instead used malachite green (MG) reagent for the detection of free phosphate (Chang et al., 2008)[S23]. DnaK (4 µM), DnaJ (0.4 µM), and GrpE (0.4 µM) were diluted in reaction buffer containing Buffer B. Reactions were made to a final volume of 30 µL in a 96-well flat-bottom clear polystyrene microplate (Costar). The microplate was spun down in a Sorvall ST16 (ThermoFisher Scientific) centrifuge for 1 minute at 142 x g after the addition of each reaction component except the MG reagent. A two-fold serial dilution of each compound in DMSO was performed and added to reactions containing DnaK, resulting in a final DMSO content of 20% (v/v). Afterwards, the plate was incubated for 20 minutes at 37°C. Cofactors were then added to each reaction, which was followed by another incubation for 20 minutes at 37°C. Reactions were initiated by adding ATP (100 µM) and allowed to run for 30 minutes before being quenched by 30 µL of 0.05% formic acid. 60 µL of MG reagent (prepared as described above) was mixed with quenched reactions and left to incubate for 30 minutes at room temperature. Absorbance at 630 nm was measured using an Agilent Biotek Synergy H1 plate reader. Absorbance was converted to the concentration of phosphate produced using a standard curve generated with potassium phosphate. To calculate "Inhibition of Activation", reactions containing DnaK, DnaJ, GrpE, and compound were normalized to control reactions containing DnaK only with 20% DMSO (100%) and reactions containing DnaK-cofactors with 20% DMSO (0%). Half-maximal inhibitory concentrations (IC50) for each compound's inhibition of ATPase activation were calculated in GraphPad Prism using the function logEC50 = logECF − (1/HillSlope)log(F/100-F) and Y = Bottom + (Top-Bottom)/(1+10^((LogEC50-X)HillSlope)). To determine 50% inhibition values, F was set to 50.

Cofactor titration experiments were conducted similarly to compound dose-response experiments with slight adjustments. 100 µM compound dissolved in DMSO was added to

reactions (10% DMSO) containing 4 µM DnaK. DnaJ concentration was varied (1.6, 0.8, 0.4, and 0.2 µM) while GrpE concentration was kept constant at 0.4 µM. All reactions were initiated by adding ATP (100 µM), run for 30 minutes at 37°C, and quenched with 30 µL of 0.05% formic acid. Absorbance measurements were obtained using malachite green detection as described above. To calculate "% Inhibition", reactions containing DnaK-DnaJ-GrpE and compound were normalized to control reactions containing DnaJ and GrpE only with 10% DMSO (100%) and reactions containing DnaK-DnaJ-GrpE in 10% DMSO (0%). It should be noted that in some experiments, addition of GrpE showed only weak stimulation of DnaK ATPase activity compared to addition of DnaJ.

Fluorescence polarization (FP) assays with *E. coli* DnaK and hit compounds

All FP experiments were adapted from published protocols (Rossi and Taylor, 2011[S25]; Ricci and Williams, 2008[S26]; Moerke, 2009[S27]). For all experiments, reactions were conducted in a 96-well flat-bottom black plate (Corning) at a final volume of 50 µL (10% DMSO) and read using an Agilent Biotek Synergy H1 plate reader (excitation and emission wavelength settings at 485/20 and 528/20 nm, respectively). Measured polarization values for each reaction were plotted using Graphpad Prism.

For saturation binding assays, *E. coli* DnaK, diluted in PBS containing 2 mM $MgCl_2$ and 0.1% Pluronic-127, was titrated into 25 nM FITC-HLA (FITC-$\beta A\beta A$-RENLRIALRY)[S5–S7] probe. The FITC-labeled HLA peptide (HLA = RENLRIALRY) was synthesized by standard solid-phase peptide synthesis using FITC dye dissolved in DMSO[S5–S7]. The sealed plate was rocked (200 rpm) for t=60 minutes at room temperature before being read. For compound competition assays, each compound dissolved in DMSO (100 µM final concentration) was added to reactions containing DnaK (1.5 µM) and FITC-HLA probe (25 nM). The plate was rocked and read as described for the saturation binding assay. For background polarization experiments, compound EG31 alone was titrated into 25 nM FITC-HLA probe

with no protein present. The plate was rocked and read as described above.

Co-sedimentation experiments

E. coli DnaK (4 µM) and compound (200 µM) were combined in ATPase assay reaction buffer (50 mM HEPES, pH 7.5, 2 mM $MgCl_2$, 0.01% Tween-20, 20% DMSO) to achieve a final reaction volume of 100 µL. Reactions were assembled in 0.65 mL Eppendorf tubes and incubated at 37ºC for 20 minutes. After incubation, reactions were centrifuged for 30 minutes (15493 x g, 25ºC). The supernatant was separated from the pellet immediately after spinning[S4]. The remaining pellet was resuspended in 10 µL of ATPase assay reaction buffer. Gel samples were created by combining 10 µL of supernatant or resuspended pellet with 10 µL of 2x Laemmli buffer. Band intensities were determined on ImageJ using the Analyze → Gels → Plot Lanes feature. The area under the curve of each intensity peak was taken as the band intensity for each lane.

Thermal Stability Assays

To explore the small molecules binding on protein structure stability, we deployed the thermal shift assay to measure protein melting temperature (Tm). In the initial tagless nano-DSF experiments[S28,S29], we used 14.1 µM of *E. coli* DnaK and 100µM-1mM compounds (concentration depended on individual DMSO solubilities) in 25mM Trizma-base (ph7.2), 400mM NaCl, 5%glycerol, 2mM $MgCl_2$ (DSF storage buffer), with a final volume 20 µL. 10%DMSO was used as a control. The mixture was added to capillary tubes and incubated in NanoTemper Prometheus Panta under continuous ramp mode in a starting from 25°C to 90°C, to use intrinsic fluorescence as a function of temperature to measure the unfolding of protein upon exposure of Trp (or Tyr). The melting temperature (Tm) was defined as the temperature at which the first derivative (dF/dT) of the 350 nm fluorescence signal reached its maximum. A DMSO-only protein sample served as the control.

To evaluate whether EG35 and EG36 exhibit any off-target effects on *M. tb* HtpG-a

bacterial homolog of the HSP90 family of chaperones[S30], nanoDSF assays were performed using 5 µM HtpG. Geldanamycin (GA) was included, as it is a well-known inhibitor of HSP90[S1]. ATP was used as a positive control. Samples containing 200 µM GA, 100 µM EG35, 100 µM EG36, or 100 µM ATP were prepared with 5 µM HtpG in buffer composed of 25 mM Tris(hydroxymethyl)aminomethane (Tris-base, pH 7.6), 400 mM NaCl, and 5 % glycerol with a final volume of 20 µL. All compounds were diluted in DMSO and compared to a DMSO only control.

For dye-based DSF assays[S31], 2.5 µM *E. coli* DnaK and 500, 250, 125, 62.5, 31.25 µM compounds(final concentration, stock dissolved in DMSO) were prepared in DSF storage buffer. SYPRO Orange dye (5000x, corresponding to 10mM) was diluted to 40x using DSF buffer[S31]. Then 40X dye final concentration was added to each sample resulting in a final concentration of 4X. Reaction solutions in a 96 well plate were heated up from 25°C to 90°C with an increment at 0.5 °C per cycle. The temperature at the maximum absolute number of negative first derivative (Y=-$\Delta RFU/\Delta Temperature$)is taken as melting point of the protein[S31].

Sequence alignment and domain annotation of *E. coli* DnaK with *M. tb* DnaK

Amino acid sequences for *E. coli* DnaK (UniProt entry: P0A6Y8) and *M. tb* DnaK (UniProt entry: P9WMJ9) were aligned using the multiple sequence alignment tool in Clustal Omega. Our NBD truncation of *M. tb* DnaK consisted of residues M1 to E359[S7]. The flexible DnaK linker region was annotated using previously reported residue ranges[S32].

ATPase experiments with *M. tb* DnaK NBD (M1 to E359)

All reactions (10 uL final volume) were performed in an opaque 384-well microplate (Greiner). The microplate was spun down in a Sorvall ST16 (ThermoFisher Scientific) centrifuge for 1

minute at 142 x g prior to each incubation step. DnaK NBD (10 uM) and compounds (200 uM) were combined in reaction buffer (1x PBS, 2 mM $MgCl_2$, 0.01% Tween-20, 20% DMSO) and incubated for 20 minutes at 37°C. Reactions were then initiated with ATP (500 uM) and incubated for 1 hour at 37°C. To quench reactions, 10 uL of 0.05% formic acid was added to each well. For ATP quantification, 20 uL of Kinase-Glo Max (Promega) reagent was added to quenched reactions and the plate was incubated for 10 minutes at room temperature with rocking (200 rpm). Endpoint luminescence was measured using an MD FlexStation3 plate reader. Raw luminescence values were converted to ATP (uM) remaining using an ATP standard curve. Resulting data was analyzed using GraphPad Prism.

Expression and purification of *M. tb* HtpG

Protein expression and purification of His6-tagged N-terminal HtpG were adapted from previously reported protocols[S12]. An overexpression plasmid for His-HtpG was transformed into BL21(DE3) competent cells. A 5 mL overnight culture of cells was used to inoculate 500 mL LB, supplemented with 25 µg/mL kanamycin. Cells were grown with shaking at 37°C to OD600   0.2. The temperature was lowered to 18°C and cells were induced with 0.01 mM IPTG (isopropyl-β-D-thiogalactoside) for 20 hours. Cells were harvested by centrifugation (3100 × g, 10 min, 4°C) and pellets were stored at -80°C. Cell pellets were resuspended in 15 mL of Buffer A and supplemented with 100 g/mL lysozyme. The resuspended pellet was rocked for 1 hour at 4°C and lysed by high-pressure homogenization. Cellular debris was removed from the lysate by ultracentrifugation (Beckman Coulter Allegra X-15R, 10956 x g, 40 min, 4°C). The remaining supernatant was rocked with 15 mL of washed Ni-NTA agarose resin (Qiagen) and 2 mM imidazole for 1 hour at 4°C. The resin was washed 5 times with 25 mL of 30 mM imidazole in Buffer A. His-HtpG was eluted off the column with 15 mL of 200 mM imidazole in Buffer A followed by 15 mL of 300 mM imidazole in Buffer A. Elutions were dialyzed against 2 L of Buffer A at 4°C using using Snakeskin Dialysis Tubing (Thermo Scientific, 10K MWCO, 35 mm). Dialyzed protein was concentrated to 500 µL

using a 10 kDa MWCO Amicon Ultra Centrifugal Filter Device (Millipore) at 4°C. Protein concentration was determined using by the DC protein assay (Bio-Rad) using BSA (bovine serum albumin) as the standard. Protein aliquots were flash frozen in N2(l) and stored at -80°C.

ATPase experiments with *M. tb* HtpG

All reactions (10 µL final volume) were performed in an opaque 384-well microplate (Greiner). The microplate was spun down in a Sorvall ST16 (ThermoFisher Scientific) centrifuge for 1 minute at 142 x g prior to each incubation step. Compounds were diluted in DMSO and were made as 10x stocks. HtpG (10 uM) and compounds (200 uM final concentration) were combined in reaction buffer (1x PBS, 2 mM $MgCl_2$, 0.01% Tween-20, 20% DMSO) and incubated for 20 minutes at 30°C. Reactions were then initiated with ATP (2 mM) and incubated for 2 hours at 30°C. To quench reactions, 10 µL of 0.05% formic acid was added to each well. For ATP quantification, 20 µL of Kinase-Glo Max (Promega) reagent was added to quenched reactions and the plate was incubated for 10 minutes at room temperature with rocking (200 rpm). Endpoint luminescence was measured using an MD FlexStation3 plate reader. Raw luminescence values were plotted using GraphPad Prism.

References

(S1) Dey, A.; Cederbaum, A. I. Geldanamycin, an inhibitor of Hsp90, potentiates cytochrome P4502E1-mediated toxicity in HepG2 cells. *The Journal of pharmacology and experimental therapeutics* **2006**, *317*, 1391–1399.

(S2) Lam, H. Y. I.; Guan, J. S.; Ong, X. E.; Pincket, R.; Mu, Y. Protein language models are performant in structure-free virtual screening. *Briefings in Bioinformatics* **2024**, *25*, bbae480.

(S3) Pyo, S. M.; Hespeler, D.; Keck, C. M.; Müller, R. H. Dermal miconazole nitrate nanocrystals–formulation development, increased antifungal efficacy & skin penetration. *International journal of pharmaceutics* **2017**, *531*, 350–359.

(S4) McGovern, S. L.; Helfand, B. T.; Feng, B.; Shoichet, B. K. A specific mechanism of nonspecific inhibition. *Journal of medicinal chemistry* **2003**, *46*, 4265–4272.

(S5) Riggs, J.; Seiwald, R. J.; Burckhalter, J.; Downs, C. M.; Metcalf, T. Isothiocyanate compounds as fluorescent labeling agents for immune serum. *The American journal of pathology* **1958**, *34*, 1081.

(S6) Haug, M.; Schepp, C. P.; Kalbacher, H.; Dannecker, G. E.; Holzer, U. 70-kDa heat shock proteins: specific interactions with HLA-DR molecules and their peptide fragments. *European journal of immunology* **2007**, *37*, 1053–1063.

(S7) Hosfelt, J.; Richards, A.; Zheng, M.; Adura, C.; Nelson, B.; Yang, A.; Fay, A.; Resager, W.; Ueberheide, B.; Glickman, J. F.; others An allosteric inhibitor of bacterial Hsp70 chaperone potentiates antibiotics and mitigates resistance. *Cell Chemical Biology* **2022**, *29*, 854–869.

(S8) Palleros, D.; Reid, K.; McCarty, J.; Walker, G.; Fink, A. DnaK, hsp73, and their

molten globules. Two different ways heat shock proteins respond to heat. *Journal of Biological Chemistry* **1992**, *267*, 5279–5285.

(S9) Montgomery, D.; Jordan, R.; McMacken, R.; Freire, E. Thermodynamic and structural analysis of the folding/unfolding transitions of the Escherichia coli molecular chaperone DnaK. *Journal of molecular biology* **1993**, *232*, 680–692.

(S10) Abramson, J.; Adler, J.; Dunger, J.; Evans, R.; Green, T.; Pritzel, A.; Ronneberger, O.; Willmore, L.; Ballard, A. J.; Bambrick, J.; others Accurate structure prediction of biomolecular interactions with AlphaFold 3. *Nature* **2024**, *630*, 493–500.

(S11) Passaro, S.; Corso, G.; Wohlwend, J.; Reveiz, M.; Thaler, S.; Ram Somnath, V.; Getz, N.; Portnoi, T.; Roy, J.; Stark, H.; others Boltz-2: Towards Accurate and Efficient Binding Affinity Prediction. *BioRxiv* **2025**, 2025–06.

(S12) Harnagel, A.; Lopez Quezada, L.; Park, S. W.; Baranowski, C.; Kieser, K.; Jiang, X.; Roberts, J.; Vaubourgeix, J.; Yang, A.; Nelson, B.; others Nonredundant functions of Mycobacterium tuberculosis chaperones promote survival under stress. *Molecular microbiology* **2021**, *115*, 272–289.

(S13) Sorkun, M. C.; Khetan, A.; Er, S. AqSolDB, a curated reference set of aqueous solubility and 2D descriptors for a diverse set of compounds. *Scientific data* **2019**, *6*, 143.

(S14) Fang, Y.; Zhang, Q.; Zhang, N.; Chen, Z.; Zhuang, X.; Shao, X.; Fan, X.; Chen, H. Knowledge graph-enhanced molecular contrastive learning with functional prompt. *Nature Machine Intelligence* **2023**, 1–12.

(S15) Zhu, T.; Cao, S.; Su, P.-C.; Patel, R.; Shah, D.; Chokshi, H. B.; Szukala, R.; Johnson, M. E.; Hevener, K. E. Hit identification and optimization in virtual screening: Practical recommendations based on a critical literature analysis: Miniperspective. *Journal of medicinal chemistry* **2013**, *56*, 6560–6572.

(S16) Tran-Nguyen, V.-K.; Jacquemard, C.; Rognan, D. LIT-PCBA: an unbiased data set for machine learning and virtual screening. *Journal of chemical information and modeling* **2020**, *60*, 4263–4273.

(S17) Mysinger, M. M.; Carchia, M.; Irwin, J. J.; Shoichet, B. K. Directory of useful decoys, enhanced (DUD-E): better ligands and decoys for better benchmarking. *Journal of medicinal chemistry* **2012**, *55*, 6582–6594.

(S18) Bauer, M. R.; Ibrahim, T. M.; Vogel, S. M.; Boeckler, F. M. Evaluation and optimization of virtual screening workflows with DEKOIS 2.0–a public library of challenging docking benchmark sets. *Journal of chemical information and modeling* **2013**, *53*, 1447–1462.

(S19) Victoria-Muñoz, F.; Menke, J.; Sanchez-Cruz, N.; Koch, O. Efficient decoy selection to improve virtual screening using machine learning models. *Journal of Cheminformatics* **2025**, *17*, 165.

(S20) Needleman, S. B.; Wunsch, C. D. A general method applicable to the search for similarities in the amino acid sequence of two proteins. *Journal of molecular biology* **1970**, *48*, 443–453.

(S21) Rogers, D.; Hahn, M. Extended-connectivity fingerprints. *Journal of chemical information and modeling* **2010**, *50*, 742–754.

(S22) Bajusz, D.; Rácz, A.; Héberger, K. Why is Tanimoto index an appropriate choice for fingerprint-based similarity calculations? *Journal of cheminformatics* **2015**, *7*, 20.

(S23) Chang, L.; Bertelsen, E. B.; Wisén, S.; Larsen, E. M.; Zuiderweg, E. R.; Gestwicki, J. E. High-throughput screen for small molecules that modulate the ATPase activity of the molecular chaperone DnaK. *Analytical biochemistry* **2008**, *372*, 167–176.

(S24) Richards, A.; Yawson, G. K.; Nelson, B.; Lupoli, T. J. Complementary protocols to evaluate inhibitors against the DnaK chaperone network. *STAR protocols* **2022**, *3*, 101381.

(S25) Rossi, A. M.; Taylor, C. W. Analysis of protein-ligand interactions by fluorescence polarization. *Nature protocols* **2011**, *6*, 365–387.

(S26) Ricci, L.; Williams, K. P. Development of fluorescence polarization assays for the molecular chaperone Hsp70 family members: Hsp72 and DnaK. *Current chemical genomics* **2008**, *2*, 90.

(S27) Moerke, N. J. Fluorescence polarization (FP) assays for monitoring peptide-protein or nucleic acid-protein binding. *Current protocols in chemical biology* **2009**, *1*, 1–15.

(S28) Real-Hohn, A.; Groznica, M.; Löffler, N.; Blaas, D.; Kowalski, H. nanoDSF: in vitro label-free method to monitor picornavirus uncoating and test compounds affecting particle stability. *Frontiers in Microbiology* **2020**, *11*, 1442.

(S29) Magnusson, A. O.; Szekrenyi, A.; Joosten, H.-J.; Finnigan, J.; Charnock, S.; Fessner, W.-D. nanoDSF as screening tool for enzyme libraries and biotechnology development. *The FEBS journal* **2019**, *286*, 184–204.

(S30) Berisio, R.; Barra, G.; Napolitano, V.; Privitera, M.; Romano, M.; Squeglia, F.; Ruggiero, A. HtpG—A Major Virulence Factor and a Promising Vaccine Antigen against Mycobacterium tuberculosis. *Biomolecules* **2024**, *14*, 471.

(S31) Wu, T.; Hornsby, M.; Zhu, L.; Yu, J. C.; Shokat, K. M.; Gestwicki, J. E. Protocol for performing and optimizing differential scanning fluorimetry experiments. *STAR protocols* **2023**, *4*, 102688.

(S32) Bertelsen, E. B.; Chang, L.; Gestwicki, J. E.; Zuiderweg, E. R. Solution conformation

of wild-type E. coli Hsp70 (DnaK) chaperone complexed with ADP and substrate. *Proceedings of the National Academy of Sciences* **2009**, *106*, 8471–8476.
